# Chloroquine Overcomes Chemotherapy Resistance and Suppresses Cancer Metastasis by Eradicating Dormant Cancer Cells

**DOI:** 10.1038/s41419-025-08304-6

**Published:** 2025-12-10

**Authors:** Marina A. Mikeladze, Liubov S. Kuznetcova, Elena Y. Komarova, Margarita A. Galcheva, Vladimir F. Lazarev, Lev A. Khamaev, Maria A. Konanova, Yana A. Gladova, Anna B. Danilova, Boris A. Margulis, Bashar A. Alhasan, Irina V. Guzhova

**Affiliations:** 1 Institute of Cytology of Russian Academy of Sciences, Tikhoretsky prospect, 4, S.Petersburg, Russia; 2 N.N.Petrov Scientific Medical Research Center of Oncology, Leningradskaya str, 68, Pesochny, S.Petersburg, Russia

**Keywords:** Cell death, Autophagy

## Abstract

Cancer cell resistance and tumor relapse remain major challenges in cancer treatment. Chloroquine, an FDA-approved antimalarial drug currently undergoing clinical trials for various cancers, has emerged as a promising candidate for combination therapy with conventional anticancer agents. In this study, we demonstrate that in patients-derived osteosarcoma cells who had undergone multiple chemotherapy treatments, as well as in murine colorectal cancer cells, administration of standard chemotherapeutic agents induces autophagy, which likely serves as a cytoprotective mechanism promoting therapy resistance in at least of part of tumor population. Incorporating chloroquine into the treatment regimen effectively suppressed autophagy, significantly enhancing osteosarcoma cell death in both 2D and 3D models while simultaneously reducing cell proliferation and migration capacity. In an orthotopic in vivo model of colorectal cancer, the combination of chloroquine and oxaliplatin not only impaired tumor growth but also prevented metastatic dissemination and inhibited the formation of metastasis. Notably, comparative analyses of proliferating and dormant tumor cell populations revealed that chloroquine exerts preferential cytotoxicity toward dormant cancer cells. This suggests a dual therapeutic advantage, wherein cytostatic agents primarily eliminate proliferating cells, while chloroquine specifically eradicates dormant cancer cells, which are often implicated in tumor recurrence. Collectively, these findings highlight the potential of autophagy inhibition to enhance the chemotherapy efficacy and suggest chloroquine-based combination therapy as a promising strategy for suppressing tumor growth and metastasis, ultimately improving treatment outcomes in cancer patients.

## Introduction

Osteosarcoma (OS) is a highly aggressive malignant tumor originating from bone tissue, characterized by limited treatment options and poor prognosis [1]. This neoplasm arises primarily from mesenchymal osteoblast precursor cells [2] and is predominantly localized in long tubular bones, with rare occurrences in the spine, pelvis, and sacral regions [3].

Colorectal cancer (CRC) ranks as the third most prevalent cancer globally, with an estimated 1.1 million new cases annually, and is the second leading cause of cancer-related mortality [4]. Although advancements in treatment have improved survival rates, metastatic colorectal cancer remains a lethal condition, with a 5-year survival rate of approximately 14% [5]. Metastasis occurs in 15–30% of patients, and 20–50% of those initially diagnosed with localized disease eventually develop metastatic spread. The liver is the most common site of metastasis, followed by the lungs, peritoneum, and distant lymph nodes [6].

Acquired drug resistance is a form of resistance that develops during the treatment of tumors that initially exhibit high sensitivity to therapeutic agents. This resistance arises due to the emergence of genetic mutations or the activation of adaptive cellular mechanisms [7]. Additionally, acquired resistance can lead to cross-resistance, where tumors become resistant to chemotherapeutic agents with distinct mechanisms of action, even to those not previously administered to the patient. Consequently, addressing drug resistance is critical for improving outcomes in cancer therapy [8].

Autophagy is a fundamental cellular process that facilitates the degradation and recycling of damaged organelles, protein aggregates, and other cytoplasmic components through the lysosomes. This process is essential for cellular homeostasis, adaptation to nutrient deprivation, immune regulation, and facilitation of apoptosis. Autophagy is classified into three major forms: macroautophagy, microautophagy, and chaperone-mediated autophagy [9]. Among which, macroautophagy is the most studied, whereby cellular components are sequestered within double-membraned autophagosomes, which subsequently fuse with lysosomes to form autophagolysosomes for degradation. This process is regulated by autophagy-related genes (ATGs) and plays a role in the turnover of organelles such as mitochondria, the endoplasmic reticulum, and ribosomes, as well as proteins, lipids, and RNA [10].

As tumors grow, their increasing metabolic demands often exceed the capacity of the local vasculature, leading to hypoxia and nutrient deprivation in poorly perfused tumor regions, known as necrotic foci. In response to these stress conditions, tumor cells undergo metabolic reprogramming, which frequently involves the activation of autophagy that enhances nutrient recycling and provides metabolic plasticity, enabling tumor cells to survive under stress [11]. The harsh conditions within the tumor microenvironment also facilitate the development of metastases [12]. The efficiency of colonization during the later stages of metastasis is influenced by the rate of micrometastasis formation, which depends on the ability of tumor cells to adapt to novel stromal interactions. Growing evidence highlights the critical role of autophagy in the metastatic cascade [13], as it supports the survival of dormant tumor cells [14], circulating tumor cells [15], and tumor stem cell (CSC) subpopulations, which are key mediators of invasion and treatment resistance [16].

Given the critical role of autophagy in tumor survival and metastasis, autophagy inhibition has emerged as a promising therapeutic strategy. Chloroquine (CQ) and its derivative hydroxychloroquine (HCQ) are well-established antimalarial agents belonging to the 4-aminoquinoline class. They are widely used for the prevention and treatment of malaria, extraintestinal amebiasis, and rheumatic diseases, as well as for managing inflammation [17]. More recently, CQ has gained attention as a potential anticancer agent, both as a monotherapy and in combination with other drugs due to its ability to inhibit autophagy and enhance tumor cell sensitivity to chemotherapy [18, 19].

The primary aim of this study was to investigate the impact of chloroquine on the resistance of osteosarcoma and colorectal cancer cells to chemotherapy. Additionally, we sought to evaluate how chloroquine influences the metastatic potential of colorectal cancer.

## Materials and Methods

### Cell lines and culture conditions

Osteosarcoma cell lines, OS921 and OS1056, were derived from patients undergoing chemotherapy at the N.N. Petrov National Medical Research Center of Oncology, Ministry of Health of the Russian Federation (St. Petersburg, Russia). These cell lines were established from lung metastases obtained during surgical procedures. The research protocol was approved by the local ethical committee, and all patients provided written informed consent prior to tissue collection. Tumor samples were preserved in compliance with the Helsinki Declaration and utilized in accordance with the Human Tissue Act of 2004.

All patients had undergone multiple surgical interventions due to disease progression. OS921 cells were derived from conventional osteogenic sarcoma in a 13-year-old female patient who had received seven cycles of EURAMOS and two cycles of ICE chemotherapy prior to sample collection. The patient passed away two and a half months after sample collection.

OS1056 cells were derived from chondroblastic osteosarcoma in a 39-year-old female patient who had undergone three lines of polychemotherapy (MAP, GemTax, and sorafenib/bevacizumab) before sample collection. The patient died nine months after sample collection.

Osteosarcoma cells were isolated from tumor samples and cultured, as previously described [20]. Cells were maintained for at least ten passages before experimentation.

Human osteosarcoma HOS cell line and human lung carcinoma A549 and H1299 cell lines were obtained from the “Collection of Vertebrate Cell Cultures,” supported by a grant from the Ministry of Education and Science of the Russian Federation (Agreement No. 075-15-2021-683).

Mouse colorectal carcinoma cells CT-26iRFP720luc (referred to as CT-26 luc) were transfected with the pHIV-iRFP720-E2A-Luc vector, as described previously [21].

All osteosarcoma cell lines were cultured at 37 °C in a humidified atmosphere of 5% CO₂, using DMEM/F12 medium supplemented with 10% fetal bovine serum (Gibco, USA), 100 U/ml penicillin G, and 0.1 mg/ml streptomycin (Capricorn Scientific, Germany). CT-26 luc, A549, and H1299 cells were maintained under similar conditions in DMEM medium with identical supplements.

Cell viability was assessed using trypan blue exclusion staining. Only cell populations with a viability of ≥95% were used for experiments. Cell counting was performed using a Luna II Cell Counter (Logos Biosystems, South Korea).

### ATG5 knockdown in osteosarcoma cells

Stable ATG5-knockdown HOS and OS921 osteosarcoma cells were generated by lentiviral transduction with pLKO.1-puro vectors expressing ATG5-targeting shRNA (sense: GGACGAATTCCAACTTGTTTC; antisense: GAAACAAGTTGGAATTCGTCC; custom-synthesized by Evrogen, Russia). Lentiviral particles were produced in HEK293FT cells (70–80% confluency) co-transfected with the shRNA construct, psPAX2 (Addgene #12260), and pMD2.G (Addgene #12259) using polyethylenimine (PEI 40 K; Servicebio, China). Medium was replaced 12 h post-transfection, and viral supernatants were harvested at 48 h and 72 h, filtered (0.22 μm), and used to transduce target cells in the presence of 8 μg/mL polybrene with spinoculation (1000 × g, 32 °C, 45 min). After 18 h, medium was replaced, and cells were subjected to puromycin selection (2 μg/mL) starting 48 h post-transduction for 14 days, with fresh selective medium replenished every 3 days. Knockdown efficiency was verified by Western blotting for ATG5.

### CT-26 Cells Stably Expressing EGFP-mCherry-LC3B

Retroviral particles were produced in HEK293FT cells (70–80% confluency) by transfection with EGFP-mCherry-LC3B (#22418), pMD2.G (#12259), and pUMVC (#8449, kindly provided by Dr. Victor Tatarskiy, Institute of Gene Biology, Russia) using polyethylenimine PEI 40 K (Servicebio, China). Viral supernatants were collected at 48 h and 72 h post-transfection, filtered (0.22 μm), and applied directly to CT-26 cells (30–50% confluency) in the presence of 8 μg/mL polybrene. Transduction was enhanced by spinoculation (1000 × g, 32 °C, 30 min). After 18 h, medium was replaced with fresh DMEM. Puromycin selection (2 μg/mL) was applied from 48 h post-transduction for 14 days, with renewal every 3 days. Stable cell populations were validated for EGFP/mCherry expression using the ZOE Fluorescent Cell Imaging System (Bio-Rad, USA) and confirmed by flow cytometry.

### Drug treatments

For osteosarcoma cells, Etoposide (Okasa Pharma, India) was administered at 1, 2.5, and 5.8 μM for 24 hours. In combination treatment, cells were pretreated with 25 μM chloroquine (CQ; Sigma, USA) for 16 h, followed by the addition of etoposide (1, 2.5, or 5.8 μM) for a subsequent 24-hour period.

For CT-26 luc cells, they were pretreated with 0.8 μM chloroquine for 16 h, followed by exposure to 10, 20, or 30 μM oxaliplatin for 24 hours (OxP; Okasa Pharma, India).

### Determination of cell viability and proliferation

Osteosarcoma cells sensitivity to etoposide was assessed using the MTT assay. HOS, OS921, and OS1056 cells were seeded in 96-well plates at a density of 20,000 cells/well. Cells were treated with 25 µM chloroquine (CQ) for 16 h, followed by the addition of etoposide at the specified concentrations. After 24 h of incubation, the medium was removed, and 100 µL of 3-(4,5-dimethylthiazol-2-yl)-2,5-diphenyltetrazolium bromide (MTT; Paneco, Russia) solution (0.5 mg/mL) was added to each well. Plates were incubated for 3–4 h, after which the MTT solution was removed, and the resulting formazan crystals were dissolved in dimethyl sulfoxide (DMSO; Biolot, Russia). Optical density was measured using a Varioskan LUX Multimode Microplate Reader (Thermo Scientific, USA). Data were presented as the percentage of viable cells relative to the control.

Cell viability, proliferation, and migration were monitored using the xCELLigence system (ACEA Biosciences, San Diego, CA, USA), which provides non-invasive, real-time, label-free measurements based on electrical impedance. Increased impedance corresponds to greater cell adhesion and growth. CT-26 luc cells were seeded into E-plates, treated first with 0.8 µM CQ, and after 16 h, 20 µM oxaliplatin (OxP) was added. The cell index was recorded continuously for 60 h. HOS, OS921, and OS1056 cells were seeded into E-plates, treated with 25 µM CQ, followed by 1 µM etoposide, and their proliferation was monitored over 50–90 h. The proliferative activity of dormant vs. growing A549 cells was compared by seeding cells into E-plate wells at 20,000 cells/well and incubating in medium containing 10%, 0.1%, or 0% FBS for 270 h to assess dormancy and growth dynamics.

### Real-time polymerase chain reaction (RT-PCR)

RT-PCR was used to assess the expression of autophagy markers, epithelial-mesenchymal transition (EMT) markers, and stemness-related genes.

Total RNA was isolated using TRIzol reagent (Thermo Fisher Scientific, USA). Reverse transcription was performed using the MMLV RT kit (Eurogen LLC, Russia) following the manufacturer’s protocol. RT-PCR reactions were conducted using a CFX96 Real-Time PCR system (BioRad, USA). DNA amplification was performed using the qPCRmix-HS SYBR kit (Eurogen LLC, Russia) according to the manufacturer’s instructions. Primers were purchased from Evrogen LLC (Moscow, Russia), and the sequences are listed in Table 1.

**Table 1:** Sequences of primers used in this study.

Gene	Forward	Reverse
ULK1	5‘-ATGATTCTACCCACGGCAAG- 3‘	5‘-CTGGAAGATGGTGATGGGTT- 3‘
Atg7	5‘-AAGCCATGATGTCGTCTTCCTAT-3‘	5‘-GCATTGATGACCAGCTTTCTCTT- 3‘
Snail1	5‘-ATGAGGAATCTGGCTGCTGC-3‘	5‘-CAGGAGAAAATGCCTTTGGA- 3‘
TWIST	5‘-TGCATGCATTCTCAAGAGGT-3‘	5‘-CTATGGTTTTGCAGGCCAGT- 3‘
Luciferase	5‘-CTGGAAGATGGTGATGGGTT- 3‘	5’-ACGAAGGTGTACATGCTTTGGA-3’
Aurka	5‘-GCTGGAGAGCTTAAAATTGCAG-3‘	5‘-TTTTGTAGGTCTCTTGGTATGTG-3‘
CD44	5‘-ACATCCTCACATCCAACACCTC-3‘	5‘-GCAGGTCTGTGACTGATGTACA-3‘
ADLH1	5‘-AGGAGCCGAATCAGAAATGTCA-3‘	5‘-CAAATCGGTGAGTAGGACAGGT-3‘
Ki67	5‘-TCCTAGGAAAACTCCAGTTGCC-3‘	5‘-AGACACTCTCTTTGAAGGCAGG-3‘
Zeb1	5‘-CTGCTGGGAGGATGACAGAAAG-3‘	5‘-GTAACTGCACAGGGAGCAAC-3‘
Vimentin	5‘-AGGCAAAGCAGGAGTCCACTGA-3‘	5‘-ATCTGGCGTTCCAGGGACTCAT-3‘
Actin	5‘- TATGTTGCCCTAGACTTCGAGC -3‘	5‘- CGATAGTGATGACCTGACCGTC -3

### Determination of apoptosis and cell cycle analysis

Apoptosis in cancer cells and fibroblasts was analyzed using Annexin V/PI staining and flow cytometry. Following the indicated experimental procedures, conditioned media were collected, and cells were detached, pooled with the respective media, centrifuged, washed with cold PBS, and stained with Annexin V Alexa 647 (Thermo Fisher Scientific, USA) and propidium iodide (PI) according to the manufacturer’s protocol. Flow cytometry was performed using a CytoFlex Flow Cytometer, and data were analyzed with CytExpert 2.0 software.

For cell cycle analysis, 0.5–1 × 10^6^ cells were harversted, resuspended in 0.5 mL PBS, fixed with cold 100% ethanol, and incubated on ice for 20 minutes. After centrifugation at 1000 rpm for 5 minutes, the ethanol was removed, and the pellet was dried at room temperature. Cells were then stained with a PI-RNAase solution (50 µg/mL PI (Sigma-Aldrich, USA) and 100 µg/mL RNAse Type I-A (Biolot, Russia)) and incubated in the dark for 20 minutes. Approximately 50,000–100,000 cells were analyzed using the CytoFlex Flow Cytometer, and data were processed with CytExpert 2.0 software.

### Analysis of tumor cell motility

The motility of osteosarcoma cells was evaluated using a wound healing assay. HOS, OS921, and OS1056 cells were seeded in 12-well plates at a density of 100,000 cells/mL. Upon reaching 80% confluence, a scratch was made using a culture scraper. Cells were pre-treated with 25 µM CQ for 16 h, followed by the addition of 1, 2.5, or 5.8 µM etoposide for 24 h. Wound healing was monitored for 48 h using a JuLI Stage microscope, and images were analyzed with JuLIV 2.0 software.

For CT-26luc cells, a migration assay was performed using a CIM plate. Cells were seeded in serum-free medium in the upper chamber, while the lower chamber contained medium with 10% FBS. Cells were treated with 10 µM CQ and 20 µM OxP at the indicated concentrations and monitored for 50 h using the xCELLigence system (RTCA DP xCelligence, ACEA Biosciences, USA). Data were analyzed using RTCA Analysis Software (RTCA Software Pro, ACEA, San Diego, CA, USA).

### Colony formation assay

HOS, OS921, and OS1056 cells were seeded in 6-well plates at 200,000 cells/well and treated with 25 µM CQ and etoposide at 1, 2.5, or 5.8 µM. After 24 hours, cells were washed and reseeded at 1000 cells/well. Colonies were allowed to form for nine days, fixed with 10% formaldehyde, and stained with 0.01% crystal violet (Sigma-Aldrich, USA). Plates were scanned using a ChemiDoc system (Bio-Rad, USA), and colony numbers were quantified using ImageJ.

### Osteosarcoma spheroid model and ATP activity assay

For the spheroid model, 50 μL of 1.5% agarose in DMEM/F12 medium was added to 96-well plates. HOS, OS921, and OS1056 cells were seeded at 30,000 cells/mL in 200 μL medium and incubated for 3–7 days. After spheroid formation, treatments were applied as described. Morphological changes were assessed after 96 hours using a Zeiss Axiovert 40CFL microscope and Zeiss AxioCam ERc 5 s camera (Carl Zeiss MicroImaging GmbH, Germany).

ATP activity was measured in 3D spheroid models using the EzCount ATP Cell Assay Kit (HiMedia, USA). After treatment, spheroids were transferred to luminescence plates, and firefly luciferase was added according to the manufacturer’s instructions. ATP levels were quantified by luminescence using a Varioskan LUX Multimode Microplate Reader.

### Western blotting

Western blotting was used to evaluate autophagy markers in osteosarcoma and CT-26luc cells. HOS, OS921, and OS1056 cells were treated with 1, 2.5, or 5.8 µM etoposide, while CT-26luc cells were treated with oxaliplatin at 10, 20, or 30 µM for 24 hours. Protein lysates were prepared in RIPA buffer (50 mM Tris-HCl pH 7.5, 150 mM NaCl, 1 mM EDTA, 1 mM EGTA, 1 mM PMSF, 1% Triton X-100, 10% glycerol), centrifuged at 13,500 rpm, 4 °C for 15 min, and protein concentrations were determined using the Bradford assay. Samples were then diluted in sample buffer, separated by SDS-PAGE, transferred onto nitrocellulose membranes, and probed with antibodies against Atg5, Beclin1, LC3A/B (Cell Signaling Technology, USA), SQSTM1/p62 (Abcam, UK), and α-tubulin (Thermo Fisher, USA). Secondary anti-rabbit antibodies conjugated with peroxidase (Repertoire (St. Petersburg, Russia, # GARabHRP_100) were used, and detection was performed using SuperSignal West Femto reagent on a ChemiDoc system (Bio-Rad, USA), and band intensities were analyzed using ImageJ.

### Animals

All in vivo experimental protocols were approved by the Licensing Committee of the Institute of Cytology, Russian Academy of Sciences (Identification number: F18-00380). All procedures were conducted in compliance with relevant guidelines and regulations and are reported in accordance with the ARRIVE guidelines.

### Preparation of tumor cells for administration

On the day of the procedure, CT-26luc cells in the logarithmic growth phase harvested, centrifuged at 1500 rpm for 5 min, and resuspended in serum-free DMEM. The cell count was determined, and the appropriate number of cells per mouse was prepared in 1 mL of medium. Five minutes prior to injection, cells were centrifuged again at 1500 rpm for 5 min, and the pellet was resuspended in a 1:1 mixture of 50 μL serum-free DMEM and 50 μL Matrigel (Corning Incorporated, USA).

### Orthotopic tumor cell injection and treatment protocol

Eighty female BALB/c mice were used for the experiments. Animals were anesthetized with a mixture of Zoletil-100 (tiletamine hydrochloride and zolazepam, Virbac, France) and Rometar (xylazine hydrochloride, Bioveta, Czech Republic) diluted in saline, administered intraperitoneally at 100 µL per mouse. After confirming the absence of a corneal reflex, abdominal area was shaved, disinfected, and a small incision was made to expose the cecum. Tumor cells (1 × 10⁶ per mouse) were injected into the submucosal layer of the cecum. Throughout the procedure, internal organs were periodically moistened with saline to prevent desiccation. After tumor cell injection, the abdominal wall was sutured, and diclofenac (Atoll, Russia) was administered for postoperative analgesia.

Seven days post-inoculation, mice were randomly divided into four groups (*n* = 20 per group): Group 1 (Untreated): Received saline as a control, Group 2 (CQ): Treated with chloroquine (20 mg/kg) three times per week, Group 3 (OxP): Treated with oxaliplatin (50 mg/kg) once per week, and Group 4 (CQ+OxP): received a combination of chloroquine and oxaliplatin at the same doses and schedules as in Groups 2 and 3.

On day 30, 10 mice from each group underwent intravital bioluminescence imaging, after which they were euthanized and dissected to visually assess the presence of organ metastases. Then, the cecum, liver, lungs, and spleen were harvested for luciferase expression analysis using real-time PCR. The remaining 10 mice per group continued treatment for survival analysis.

### Intravital bioluminescence imaging

Luciferin was dissolved in DPBS (Paneco, Russia) at 30 mg/mL. Mice were injected with 100 µL of luciferin and anesthetized using isoflurane (Aerrane). Luminescence was detected using an IVIS Spectrum In Vivo Imaging System (PerkinElmer, USA) in automatic signal accumulation mode.

### RNA isolation from organs and luciferase expression analysis

Following euthanasia, the cecum, liver, lungs, and spleen were collected, rinsed in PBS, and cut into small fragments. Tissue homogenization was performed using ultrasound in ExtractRNA solution (Evrogen, Russia) at a ratio of 100 mg of tissue per 1 mL of reagent. Total RNA was then extracted according to the manufacturer’s protocol. Luciferase expression was quantified using RT-PCR using a CFX96 Real-Time PCR System (Bio-Rad, USA) with primers listed in Table 1.

### Models of cancer cells dormancy

Dormancy in HOS, OS921, and CT-26 cells was induced by transferring the cells into culture media containing 0%, 0.1%, or 1% FCS. Cells were seeded into E-plate wells and monitored using the xCELLigence system (ACEA Biosciences, San Diego, CA, USA) for five days.

HOS, OS921, and CT-26 cells were stained with the lipophilic dye PKH26 (MINI26-1KT, Sigma-Aldrich, USA, St. Louis) according to the manufacturer’s instructions and seeded into 24-well plates. Some wells were maintained in complete growth medium to allow proliferation, while others were switched to medium containing 0.1% FCS to induce dormancy. Cells were imaged using a ZOE Fluorescent Cell Imager (Bio-Rad, Hercules, California, USA) on days 1, 3, 5, and 7 following serum deprivation. The number of PKH26-positive cells was quantified by flow cytometry using a CytoFlex Flow Cytometer (Beckman Coulter, USA). Dormancy was confirmed using RT-PCR to assess the expression of markers related to proliferation, autophagy, EMT, and stemness in osteosarcoma cells, and by Western blotting for CT-26 cells.

### Statistical Analysis

Data are presented as mean ± standard error of the mean (SEM). Statistical analyses were performed using GraphPad Prism 10.4.0. The Nonparametric One-Way ANOVA with Tukey’s multiple comparison-test was used to compare groups, with statistical significance set at *p* < 0.05. Survival analysis was conducted using Kaplan-Meier curves.

## Results

### Anticancer drugs etoposide and oxaliplatin induce autophagy activation in osteosarcoma and colorectal cancer cells

Previous studies have reported that cisplatin can induce autophagy in tumor cells, which may either promote cell death or contribute to drug resistance [22]. To explore this further, we selected osteosarcoma cells, which are commonly treated with etoposide, and murine colorectal cancer cells, for which platinum-based drugs are indicated [23, 24].

**Fig. 1:**
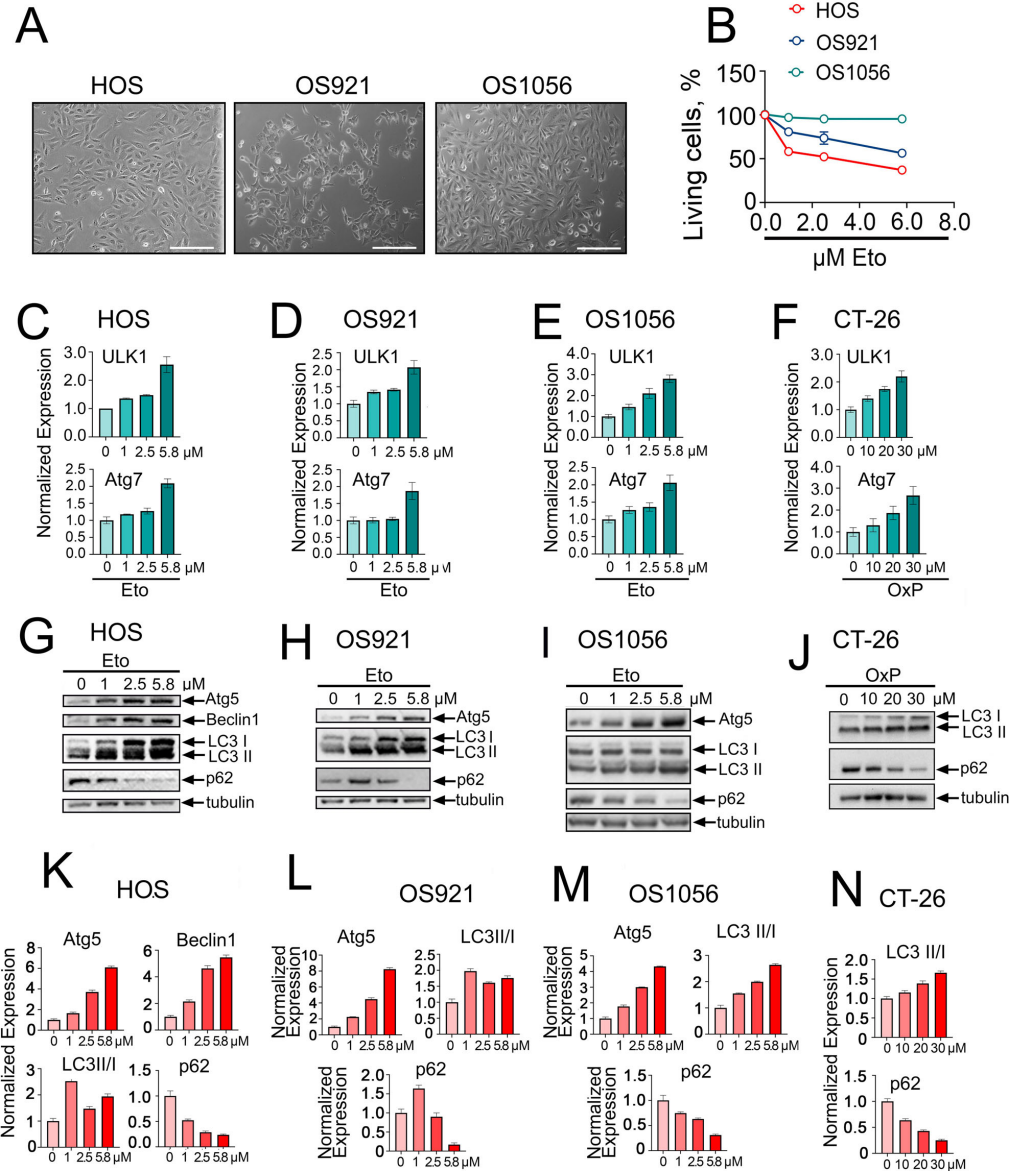
Anticancer drugs activate autophagy in osteosarcoma and colorectal cancer cells. **А** Morphology of osteosarcoma cells: established HOS cells and patient-derived OS921 and OS1056 cells. Scale bars: 200 µm; **B** MTT assay results for HOS, OS921, and OS1056 cells treated with etoposide at indicated concentrations during 48 h (*n* = 3); **C**–**F** RT-PCR data showing the expression of autophagy-related genes ULK1 and Atg7 in **C** HOS, **D** OS921, **E** OS1056, and **F** CT-26 cells treated with etoposide or oxaliplatin at indicated concentrations for 24 h; **G**, **K** Western blot analysis of autophagy markers in HOS cells treated with etoposide for 24 h and **K** quantification of protein expression normalized to Tubulin; **H**, **L** Western blot results of OS921 cells and their respective quantification; **I**, **M** Western blot results of OS1056 cells and their respective quantification; **J**, **N** Western blot results of CT-26 cells and their respective quantification.

In this study, we utilized the well-established HOS osteosarcoma cell line and two additional cell lines, OS921 and OS1056, derived from lung metastases of osteosarcoma patients who had undergone multiple chemotherapy regimens. The patient-derived cells exhibited morphology similar to HOS cells (Fig. 1A) but demonstrated significantly higher resistance to etoposide compared to HOS cells (Fig. 1B).

To evaluate the impact of etoposide and oxaliplatin on autophagy activation, we performed RT-PCR to assess the expression of ULK1 and Atg7. Osteosarcoma cell lines HOS, OS921, and OS1056 were treated with etoposide at concentrations of 1, 2.5, and 5.8 µM, while CT-26 colorectal cancer cells were treated with oxaliplatin at concentrations of 10, 20, and 30 µM. In all cell lines, we observed a dose-dependent increase in the expression of both ULK1 and Atg7 (Fig. 1C–F). Western blot analysis further confirmed autophagy activation, showing a dose-dependent increase in the levels of Atg5 and LC3-II, along with a decrease in p62 levels, a marker of autophagic flux (Fig. 1G–J). In HOS cells, we also evaluated Beclin 1 expression, which increased more than 5.5-fold following treatment with 5.8 µM etoposide (Fig. 1G, K). These findings, which focus on downstream markers of autophagy, strongly suggest that both etoposide and oxaliplatin stimulate autophagy activation in osteosarcoma and colorectal tumor cells.

### Chloroquine enhances the efficacy of etoposide in osteosarcoma and oxaliplatin in colorectal cancer

Autophagy predominantly serves as a protective cellular mechanism in cancer cells, often contributing to malignant progression and chemotherapy resistance [25]. It is also associated with cellular processes such as dormancy and senescence [26]. To investigate whether autophagy modulation would overcome chemotherapy resistance in osteosarcoma, we selected chloroquine (CQ) as an autophagy inhibitor. CQ is FDA-approved for other diseases and has demonstrated efficacy in overcoming resistance in colorectal cancer cells [27]. To determine a safe concentration of CQ for normal cells, we conducted cytotoxicity assays using dermal fibroblasts (DF2). Both CQ alone and in combination with etoposide were tested, and we found that 25 µM CQ was non-toxic to normal cells, including when combined with etoposide at various concentrations (Fig. S1). However, surprisingly, initial experiments combining CQ and etoposide simultaneously on osteosarcoma cells demonstrated no significant difference in cell death compared to etoposide treatment alone (Fig. S2).

**Fig. 2:**
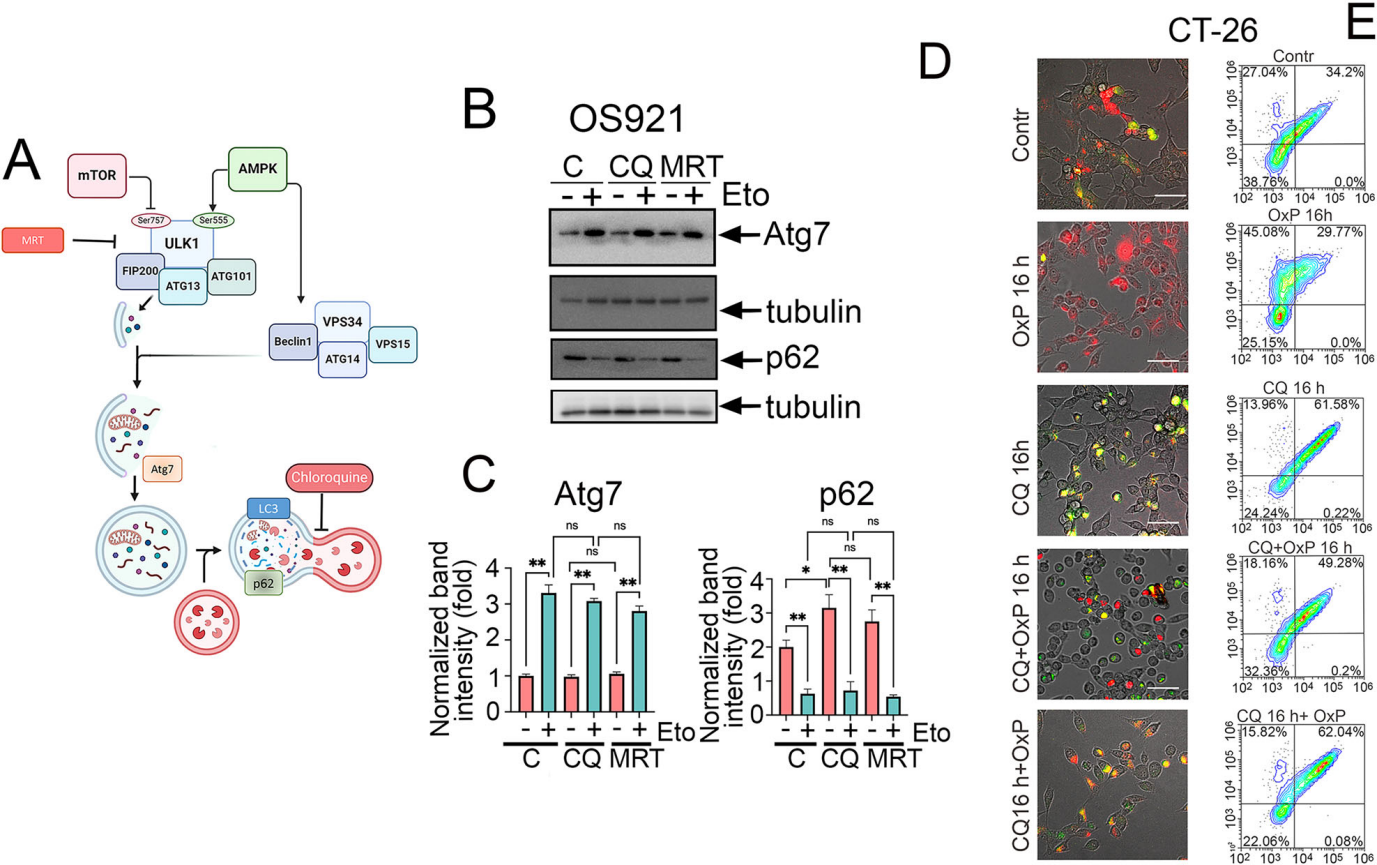
Chloroquine requires 16 hours to activate autophagy, but when used concurrently with tumor drugs, the autophagic flux is weakened. **A** Schematic mechanism of autophagy showing the sites of action of inhibitors and proteins shown on Western blot B. **B** Western blot of OS921 cells co-incubated wit 3 μM MRT68921 with 5.8 μM etoposide or 25 μM CQ with 5.8 μM etoposide for 16 h. **C** Quantification of protein expression normalized to tubulin; **B**; **D** Autophagic flux analysis in CT-26 cells expressing EGFP-mCherry-LC3B after 16-hour treatment with 25 µM CQ, 20 µM OxP, or their combinations. Representative fluorescence images were acquired using the ZOE Fluorescent Cell Imager. **E** Flow cytometric quantification of autophagic flux in CT-26 cells expressing EGFP-mCherry-LC3B following treatment with 25 µM CQ, 20 µM OxP, or their combinations as specified.

We hypothesized that this lack of efficacy may have been arised from incomplete autophagy inhibition when CQ and the chemotherapeutic agent were co-administered. To test this, we adopted an optimized regimen involving a 16-h pre-treatment with CQ prior to etoposide application, as suggested by previous studies [28, 29], and then sought to directly compare the efficacy of simultaneous versus pre-treatment strategies in inhibiting autophagy. For this purpose, we first treated OS921 osteosarcoma cells for 16 h with 5.8 μM etoposide simultaneously with either 25 μM CQ or 3 μM MRT68921, an early-stage autophagy inhibitor by suppressing ULK1, in contrast to CQ that blocks the late stage (Fig. 2A). Our results confirmed that treatment with CQ or MRT68921 alone for 16 h successfully induced p62 accumulation, verifying their inhibitory activity. However, when either inhibitor was co-administered simultaneously with etoposide, they failed to prevent p62 degradation after 16 h (Fig. 2B, C). This provides direct evidence on incomplete autophagy blockade when autophagy inhibitors are co-administrated simultaneously with chemotherapeutic agents, which may explain the initial failure to sensitize cells with simultaneous treatment.

To visually corroborate these findings across a different model, we employed CT-26 cells stably expressing the EGFP-mCherry-LC3B reporter. In this system, successful autophagic flux is indicated by a loss of EGFP signal (quenched in acidic lysosomes) while mCherry remains stable. As expected, oxaliplatin treatment for 16 h enhanced autophagic flux, shown by increased mCherry puncta and diminished EGFP signal compared to control conditions (Fig. 2D). Interestingly, and mirroring the p62 results, a 16-h CQ treatment alone effectively blocked the autophagic flux (evident by EGFP accumulation and yellow puncta), but failed to do so completely when administered simultaneously with oxaliplatin. Conversely, pre-treatment with CQ followed by oxaliplatin resulted in distinct fluorescence patterns and more pronounced autophagy blockade, characterized by intense EGFP and yellow puncta, confirming the necessity of pre-treatment for maximal autophagy inhibition (Fig. 2D).

To complement our imaging analyses with quantitative population-level assessment, we employed flow cytometry using a quadrant-based approach. This method leverages the pH-sensitive EGFP-mCherry-LC3B reporter, where enhanced autophagic flux leads to EGFP quenching in acidic autolysosomes (detected as an increase in mCherry-only cells), while flux inhibition causes accumulation of the non-degraded, dual-fluorescent protein (detected as an increase in EGFP/mCherry double-positive cells). Our quantitative flow cytometric analysis revealed distinct autophagic dynamics under the various treatment regimens. Oxaliplatin treatment significantly enhanced autophagic flux, increasing mCherry-only cells from 27% to 45% while reducing double-negative populations from 39% to 25% compared to control cells, indicating that tumor cells became more autophagic (Fig. 2E). Meanwhile, CQ monotherapy effectively suppressed autophagic flux, accumulating double-positive cells to 62% while reducing mCherry-only cells to 14%. The sequence of combination treatment proved critically important: simultaneous administration of oxaliplatin and CQ resulted in an intermediate phenotype (49% double-positive, 18% mCherry-only), suggesting that the inhibitory effect of CQ was partially mitigated by the robust flux induction by oxaliplatin. In contrast, pre-treatment with CQ prior to oxaliplatin application resulted in a profound and dominant blockade of flux, yielding a double-positive population of 62%, identical to CQ monotherapy, and a minimal mCherry-only population of 16% (Fig. 2E). Collectively, these findings demonstrate that the efficacy of autophagy inhibition is critically dependent on treatment sequence. While simultaneous administration of chloroquine with chemotherapeutic agents provides insufficient blockade of autophagic flux, pretreatment with CQ prior to chemotherapy ensures effective and complete inhibition of autophagy. This pretreatment strategy successfully prevents autophagosome-lysosome fusion and subsequent cargo degradation, thereby representing an optimized approach for therapeutic autophagy suppression in combination cancer therapy.

**Fig. 3:**
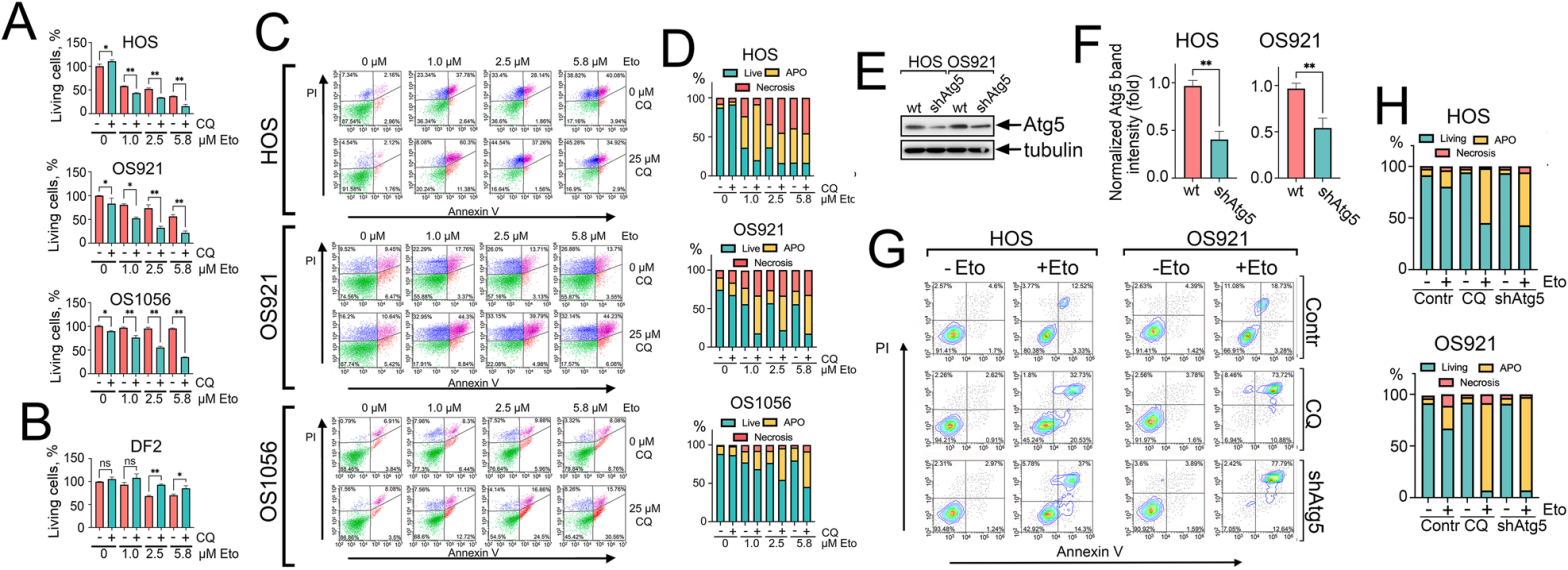
Chloroquine enhances the cytotoxic effect of etoposide on osteosarcoma cells. **A** HOS, OS921, and OS1056 cells were pre-treated with 25 µM CQ for 16 h, followed by etoposide treatment at 1, 2.5, and 5.8 µM. Cell viability was assessed 24 hours later using the MTT assay. *p < 0.05, **p < 0.0001 (*n* = 3). **B** Dermal fibroblasts (DF2) underwent the same treatment as in **C** and were analyzed using the MTT assay (*p < 0.05, **p < 0.0001; *n* = 3). **C** HOS, OS921, and OS1056 cells treated as in **A** and 48 h later were stained with Annexin V and PI, then analyzed via flow cytometry. Representative density plots are shown. **D** Quantification of viable, apoptotic, and necrotic cell populations from three independent experiments. **E** HOS and OS921 cells were generated using lentiviral transduction with shRNA targeting ATG5. Knockdown was confirmed with western blot **E** and quantification of Atg5 expression normalized to tubulin **F**; **G** HOS and OS921 cells, wt and shAtg5 were treated with 2.5 µM Eto, as well as HOSwt and OS921wt cells treated with 25 µM CQ, 16 h before Eto were stained with Annexin V and subjected to flow cytometry; **H** Quantification of viable, apoptotic, and necrotic cell populations from two independent experiments.

To confirm this, using the MTT assay, we observed a significant reduction in the viability of all three osteosarcoma cell lines following sequential treatment. This effect was particularly pronounced in patient-derived osteosarcoma cells resistant to etoposide. Specifically, in HOS cells treated with 5.8 μM etoposide alone, 37.0 ± 0.5% of cells survived, whereas pre-treatment with CQ reduced survival to 15.8 ± 1.8%. For OS921 cells, etoposide alone resulted in 56.3 ± 2.1% viability, while CQ pre-treatment reduced this to 21.6 ± 2.3%. Notably, OS1056 cells, which displayed high resistance to etoposide, exhibited 95.5 ± 0.7% survival with etoposide alone, but this dropped to 35.1 ± 0.3% with CQ pre-treatment (Fig. 3A).

Interestingly, the opposite effect was observed in dermal fibroblasts, where CQ pretreatment increased cell viability. At the highest etoposide concentration, 70 ± 1.5% of fibroblasts remained viable after etoposide alone, compared to 85.3 ± 3.1% with CQ pretreatment (Fig. 3B).

To further explore the mechanism of cell death, we performed Annexin V/PI staining followed by flow cytometry. Our results revealed that both etoposide alone and its combination with CQ induced cell death through apoptosis and necrosis, but CQ pre-treatment significantly favored and prompted apoptotic cell death in osteosarcoma cells (Fig. 3C, D).

To confirm that autophagy inhibition is primarily responsible for sensitizing osteosarcoma cells to anticancer agents, we compared the effects of genetic and pharmacological autophagy blockade on etoposide-induced cytotoxicity. We treated ATG5-knockdown HOS and OS921 cells (Fig. 3E, F) with 2.5 μM etoposide (Eto) and compared their response to that of wild-type cells pretreated with chloroquine (CQ) for 16 hours prior to Eto exposure. Our Annexin V staining and flow cytometry results demonstrate that both genetic ablation of ATG5 and pharmacological inhibition with CQ produce a similar outcome: a significant increase in etoposide-induced cell death compared to Eto alone (Fig. 3G, H).

**Fig. 4:**
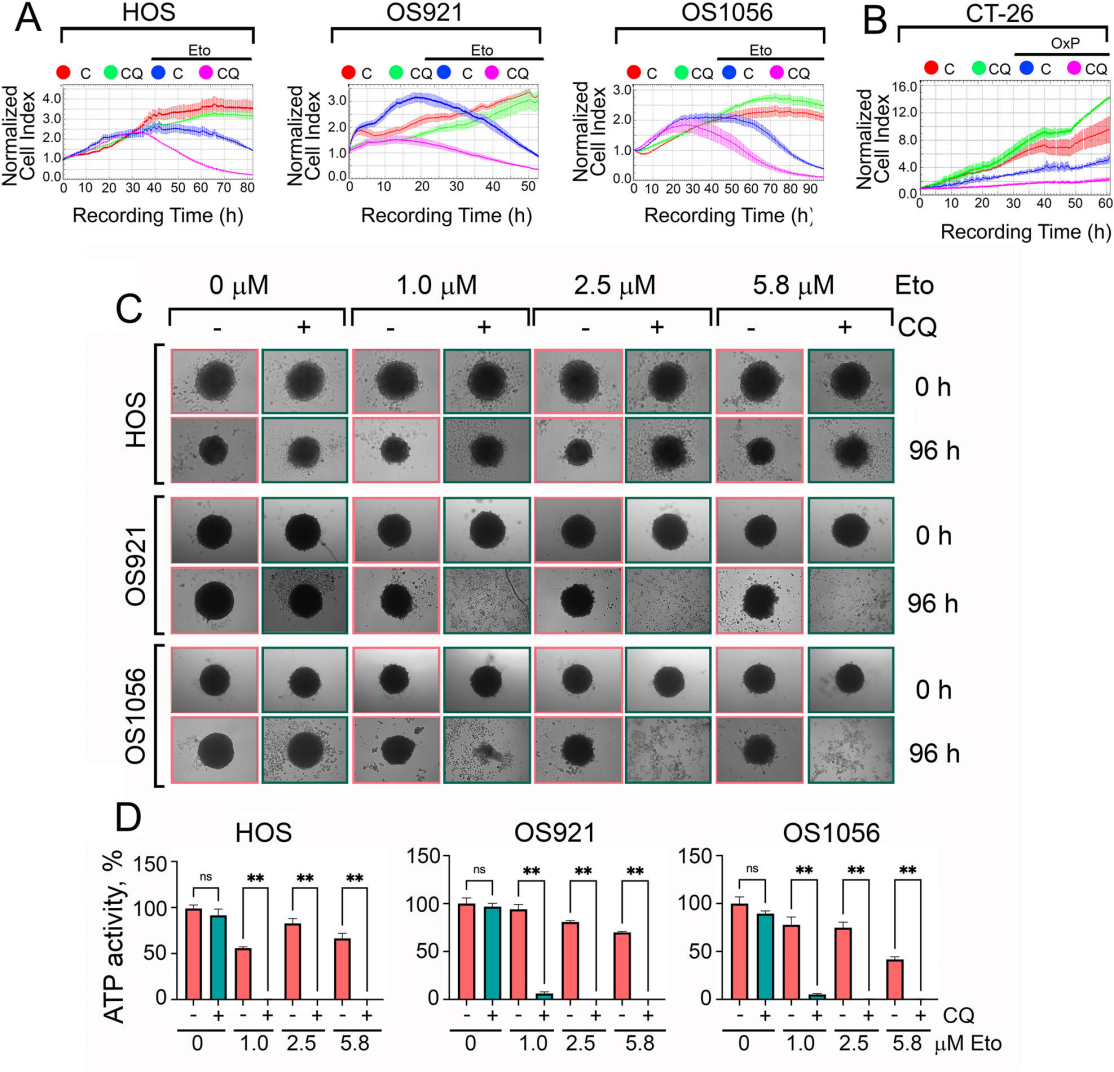
Chloroquine enhances the effect of etoposide and oxaliplatin in 2D and 3D tumor models. **A** HOS, OS921, and OS1056 cells were pre-treated with 25 μM CQ for 16 h, followed by 1 μM etoposide. Cell viability was monitored in real time using xCELLigence technology. **B** CT-26 cells were treated with 0.8 µM CQ, 20 µM OxP, or their combination and analyzed using xCELLigence technology. **C** Spheroids of HOS, OS921, and OS1056 cells were treated with 25 μM CQ, followed by 1, 2.5, or 5.8 μM etoposide after 16 h. After 96 h, spheroid morphology was assessed via microscopy. **D** ATP levels in the culture medium of spheroids treated with CQ and etoposide and their combinations. *p < 0.05, **p < 0.0001 (*n* = 3).

We further validated these findings using xCELLigence technology, which allows real-time monitoring of cell viability. In all three osteosarcoma cell lines, CQ pretreatment led to nearly complete cell death, which occurred more rapidly and efficiently than with etoposide alone (Fig. 4A). Similar results were observed in CT-26 colorectal cancer cells treated with a combination of 0.8 µM CQ and 20 µM OxP, where the cell index was more than twofold lower compared to oxaliplatin alone (Fig. 4B).

Next, we examined the effect of CQ and etoposide in a 3D spheroid model, which better mimics tumor behavior in vivo. Spheroids were treated with CQ, etoposide at 1, 2.5, or 5.8 µM, or their combination. The most pronounced effect was observed in HOS spheroids treated with 5.8 μM etoposide and CQ, where spheroids lost their defined contours and began disintegrating after 96 hours. More resistant OS921 and OS1056 spheroids exhibited complete degradation with a combination of CQ and only 1 μM etoposide (Fig. 4C).

To confirm these observations, we measured ATP levels using the EZcount™ ATP Cell Assay Kit. ATP, produced by metabolically active and viable spheroid cells, generates bioluminescence upon oxidation of D-luciferin by firefly luciferase. While CQ- and etoposide-treated HOS spheroids maintained some structural integrity, ATP activity was nearly undetectable. Similarly, ATP depletion confirmed near-total cell death in OS921 and OS1056 spheroids pre-treated with 25 μM CQ, further supporting the effectiveness of this combination therapy (Fig. 4D).

Our results demonstrate that chloroquine significantly enhances the cytotoxic effects of etoposide in osteosarcoma and oxaliplatin in colorectal cancer cells. Importantly, this combination therapy effectively overcomes chemotherapy resistance, particularly in patient-derived osteosarcoma cells, while protecting normal fibroblasts. These findings highlight the potential clinical relevance of chloroquine as an adjuvant to conventional chemotherapy.

### CQ, introduced into the chemotherapy regimen, prevents the ability of osteosarcoma and mouse colorectal cancer cells to develop metastatic phenotype in vitro and in vivo

Both osteosarcoma and colorectal cancer are highly metastatic and prone to recurrence, posing significant therapeutic challenges [30, 31]. To evaluate whether chloroquine (CQ) enhances the efficacy of chemotherapy in preventing metastasis, we assessed the impact of CQ on cell migration, epithelial-mesenchymal transition (EMT) markers, proliferative capacity from a dispersed population in vitro, as well as tumor growth and metastasis in vivo.

**Fig. 5:**
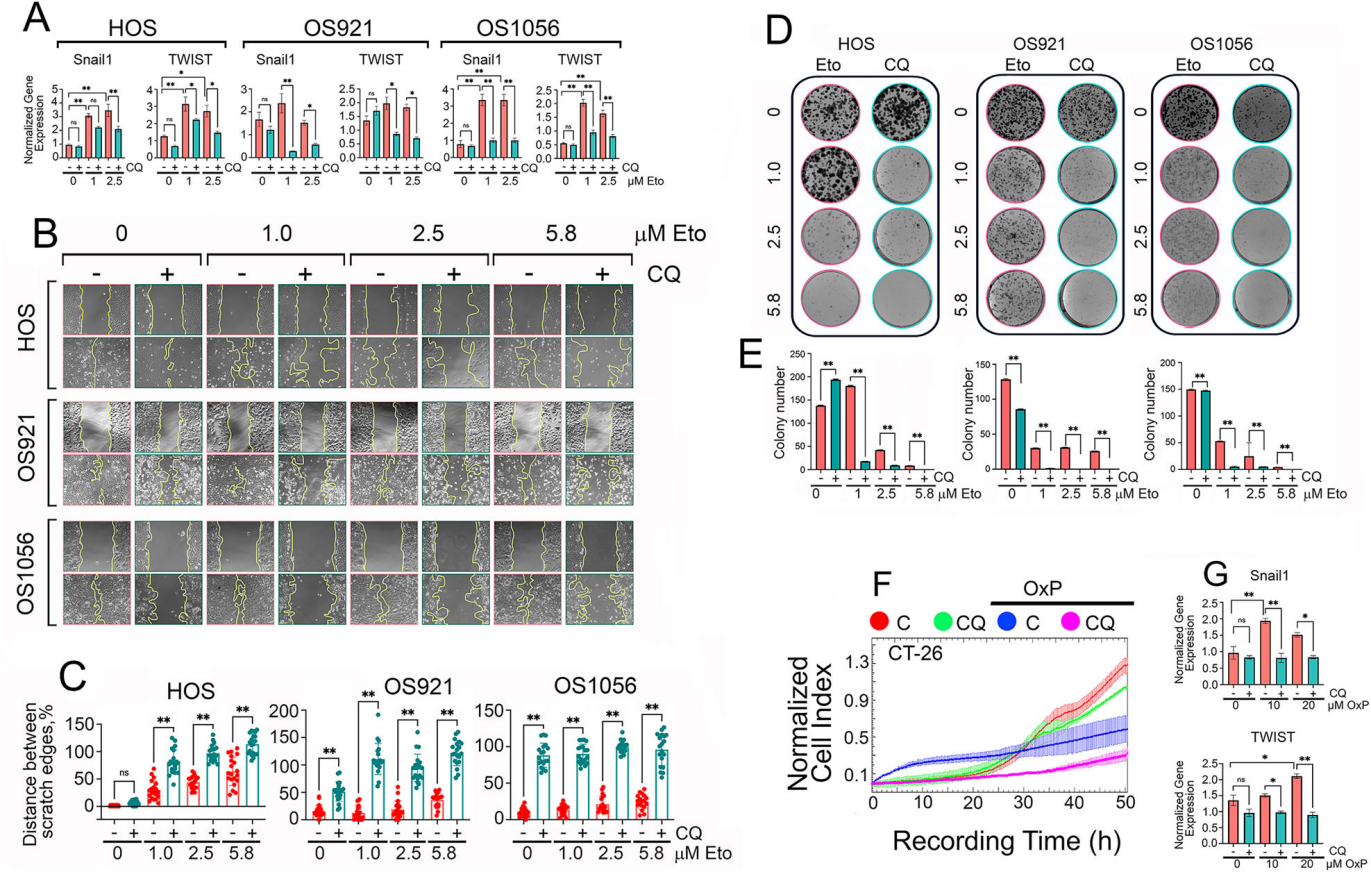
CQ in combination with anticancer drugs reduces the migratory ability of osteosarcoma and colorectal cancer cells and inhibits osteosarcoma cell proliferation from a dispersed population. **A** RT-PCR analysis of osteosarcoma cells treated with 25 µM CQ, etoposide in concentrations indicated, or their combination for 24 hours. *p < 0.05; **p < 0.005. **B** Representative images from a wound healing assay in osteosarcoma cells treated as described above. Images were taken at the start of the experiment and after 24 h. **C** Quantification of wound closure using JuLIV 2.0 software. *p < 0.05, **p < 0.0001. **D** Migration of CT-26luc colorectal cancer cells treated with CQ, OxP, or their combination, assessed by xCELLigence. **E** RT-PCR analysis of EMT markers Snail1 and TWIST in CT-26luc cells treated as in **D**. *p < 0.05, **p < 0.0001. **F** Representative images from colony formation assays in osteosarcoma cells pre-treated with 25 µM CQ, etoposide, or their combination. **G** Quantification of colony numbers using ImageJ. **p < 0.0001.

Although osteosarcoma cells are non-epithelial, CQ treatment alone did not reduced the expression of Snail1 and TWIST in OS921, OS1056, and the control HOS cell line. In contrast, etoposide alone increased the expression of both Snail1 and TWIST by two- to threefold at concentrations of 1 and 2.5 μM across all three osteosarcoma cell lines. However, co-treatment with CQ and etoposide significantly downregulated Snail1 and TWIST expression compared to etoposide alone. (Fig. 5A). In the wound healing assay, etoposide monotherapy had no substantial impact on scratch closure, while preincubation with 25 μM CQ led to a significant, reduction in wound closure compared to untreated cells and cells treated with etoposide alone within 24 hours (Fig. 5B,C). The migratory capacity of CT-26 cells was assessed using xCELLigence technology with CIM plates, which quantify cell migration through serum gradient-driven transwell migration. CQ-pretreated cells exhibited high migratory ability, similar to control cells, whereas OxP monotherapy significantly reduced migration. The combination of CQ and OxP further suppressed cell migration (Fig. 5D). Analysis of EMT marker expression in CT-26 cells revealed a pattern similar to that observed in osteosarcoma cells. Treatment with oxaliplatin (OxP) alone at concentrations of 10 and 20 μM led to a modest (approximately twofold) increase in Snail1 and TWIST expression. However, pretreatment with CQ significantly reduced the expression of both EMT markers (Fig. 5E).

The colony formation assay, which assesses the ability of tumor cells to proliferate from a dispersed population, mimicking their potential to relapse [32–34], revealed that sparsely seeded cells retained their ability to form colonies under etoposide treatment. However, preincubation with 25 μM CQ followed by etoposide (1, 2.5, or 5.8 μM) completely abolished colony formation in all three osteosarcoma cell lines (Fig. 5F, G).

To evaluate the efficacy of CQ in sensitizing tumor cells to oxaliplatin therapy in vivo, we orthotopically injected 1 × 10^6^ CT-26luc cells into the cecal mucosa of BALB/c mice. Mice were randomly divided into four groups (*n* = 20 per group): Untreated, CQ, OxP, and combination treatment (CQ+OxP). Treatment commenced on day 8 post-inoculation and continued until the experiment’s conclusion. Ten mice per group were observed for survival analysis, while the remaining ten underwent bioluminescence imaging at day 30 for metastasis assessment in the lungs, liver and spleen.

**Fig. 6:**
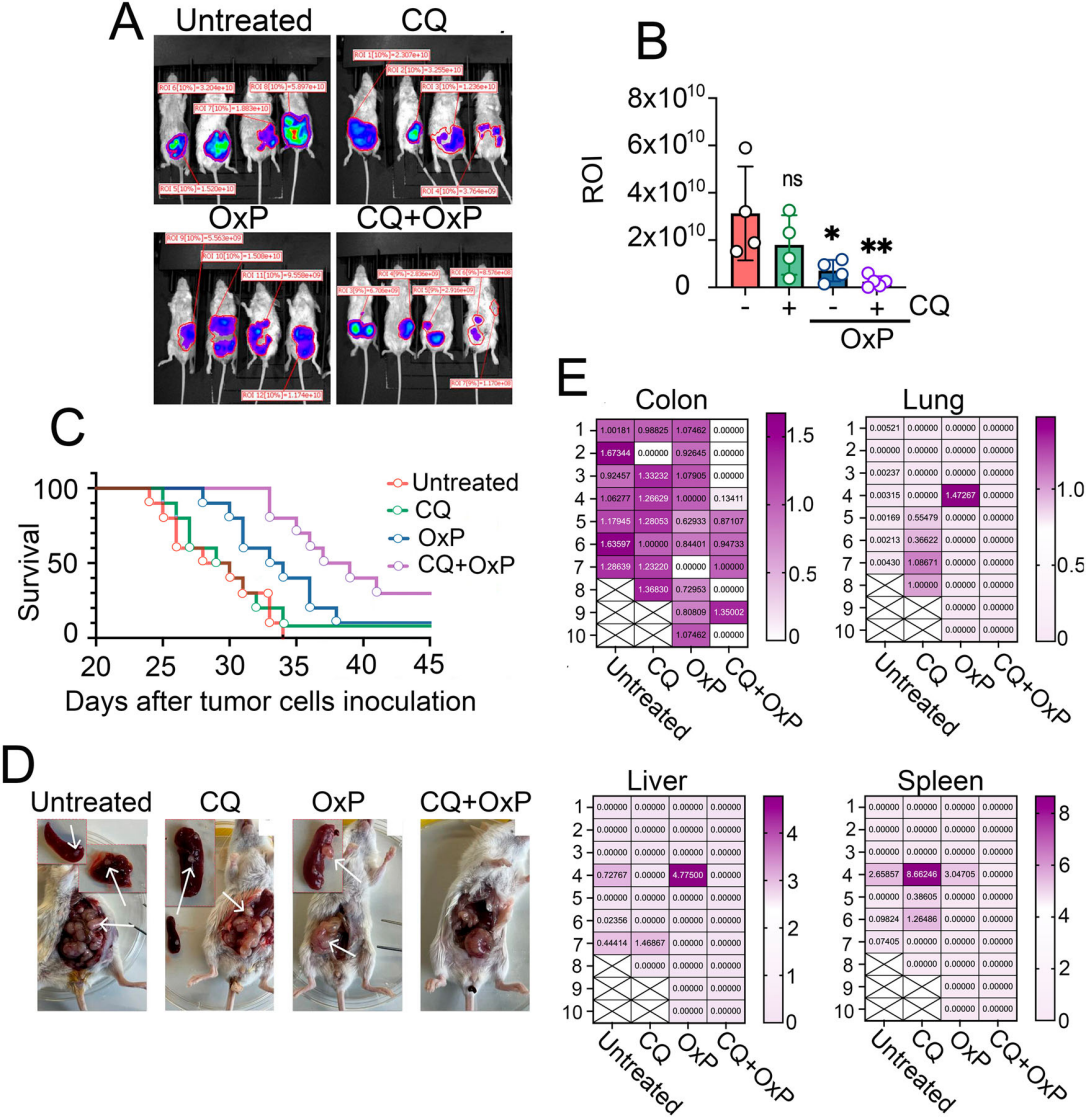
Combination of oxaliplatin and chloroquine suppresses colorectal cancer metastasis in vivo. **A** Bioluminescence imaging of mice bearing CT-26luc tumors in the colon, treated with CQ, OxP, or their combination after 30 days after tumor cells inoculation. **B** Quantification of luminescence signals (ROI) from **A**. *p < 0.05; **p < 0.01. **C** Kaplan–Meier survival curves for untreated, CQ, OxP, and CQ + OxP groups (*n* = 10 per group). **D** Visual assessment of primary tumors and metastases in organs, arrows indicate primary tumors and metastases after 30 days after tumor cells inoculation. **E** RT-PCR quantification of luciferase expression in CT-26luc cells-injected colon tissue and metastatic organs, lungs, liver, and spleen. Each luminescence value corresponds to an individual mouse. Crosses indicate mice that died before the analysis.

Bioluminescence imaging of CT-26luc tumors revealed that both CQ and oxaliplatin monotherapies slowed tumor growth. The ROI signal in the ‘CQ’ group was 1.8 × 10^10^ ± 6.2 × 10^7^, compared to 3.2 × 10^10^ ± 9.9 × 10^8^ in the ‘Untreated’ group. The ‘OxP’ group exhibited a statistically significant tumor size reduction (ROI = 7.1×10^9^ ± 2.3×10^7^, *p* < 0.005), while the ‘CQ+OxP’ combination produced the most substantial effect, reducing ROI to 2.4 × 10^8^ ± 1.0 × 10^6^ on day 30 (Fig. 6A, B).

Accordingly, tumor growth inhibition correlated with prolonged survival. The median survival time was 29 ± 1.2 days in the ‘Untreated’ group, 29.8 ± 1.2 days in the ‘CQ’ group, 33.7 ± 1.2 days in the ‘OxP’ group, and 39.7 ± 1.4 days in the ‘CQ+OxP’ group. By day 45, one mouse remained alive in both the ‘CQ’ and ‘OxP’ groups, while three mice survived in the ‘CQ+OxP’ group (Fig. 6C).

Furthermore, visual and molecular analyses were performed to assess metastasis. Visual examination of metastases revealed that animals in the ‘Untreated,’ ‘CQ,’ and ‘OxP’ groups developed metastases in the spleen and liver, while the combination group exhibited no detectable metastases in any organ (Fig. 6D). To confirm these findings, we performed RT-PCR for luciferase expression in excised organs. Three mice from the ‘Untreated’ group and two from ‘CQ’ died before the experiment. Luciferase levels at the primary tumor site were 1.3 ± 0.1 in the ‘Untreated’ group, 1.1 ± 0.2 in the ‘CQ’ group, 0.8 ± 0.1 in the ‘OxP’ group, and 0.4 ± 0.1 in the ‘CQ+OxP’ group, with the latter demonstrating significant reduction compared to the untreated control (*p* = 0.0023). Notably, luciferase was undetectable in one mouse from the ‘CQ’ and ‘OxP’ groups and in five mice from the ‘CQ+OxP’ group, suggesting remarkable successful treatment by the combination therapy (Fig. 6E).

In the ‘Untreated’ group, all but one mouse developed lung metastases, three of which also exhibited both liver and spleen metastases. In the ‘CQ’ group, four mice developed lung metastases, one had liver metastases, and two had spleen metastases. Additionally, one mouse showed a high luciferase signal in the spleen, indicating metastatic presence. In total, five out of eight mice in this group developed metastases. In the ‘OxP’ group, only one mouse developed lung metastases. Another mouse developed metastases in both the liver and spleen, leading to a total of two out of 10 mice exhibiting metastatic spread. Remarkably, in the CQ+OxP combination group, no mice developed metastases in the lung, liver, or spleen (Fig. 6E).

### Chloroquine exhibits greater toxicity toward dormant cancer cells than proliferating cells

**Fig. 7:**
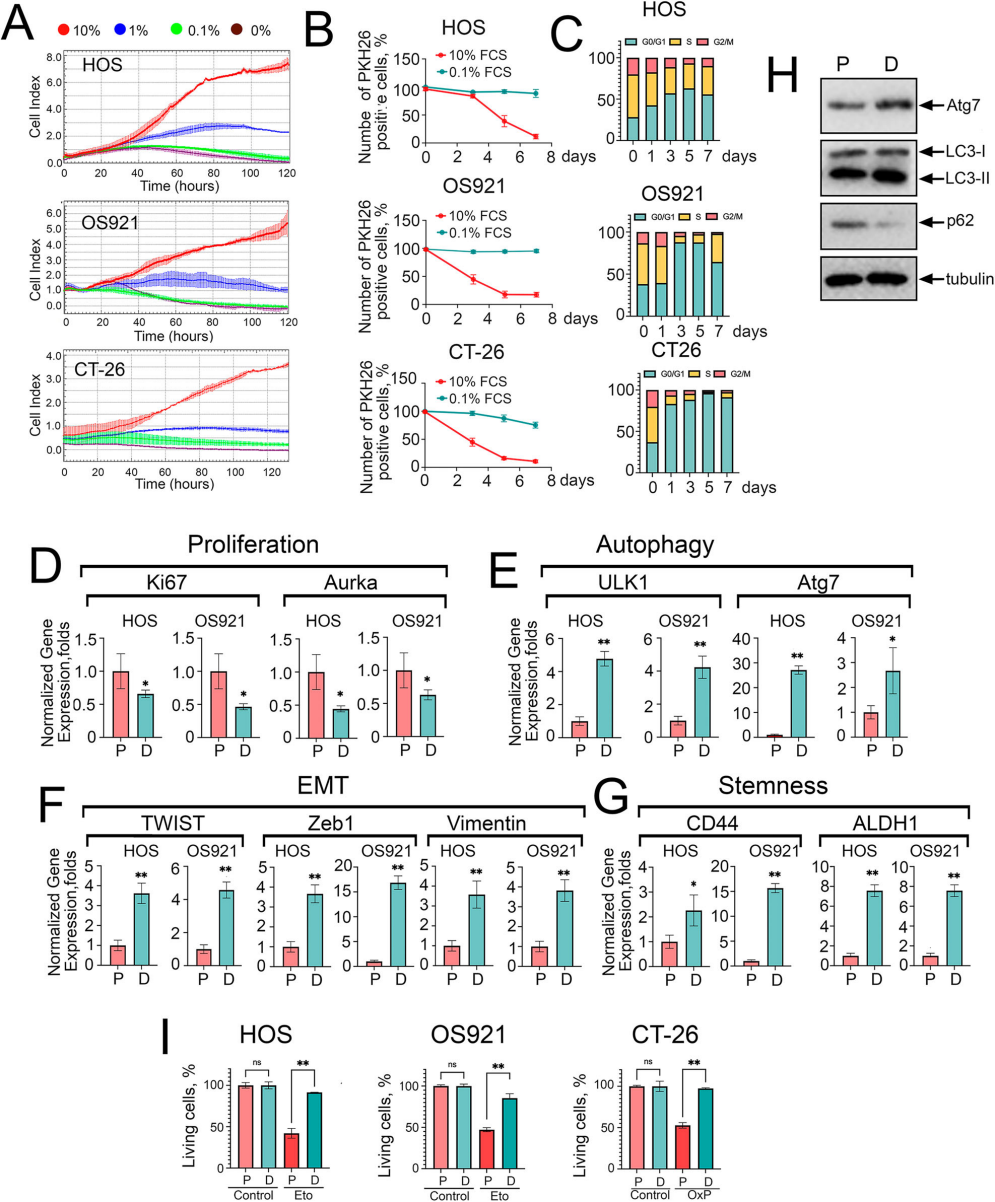
Serum deprivation in culture medium induces dormant state of tumor cells. **A** Dormancy in HOS, OS921, and CT-26 cells was induced by transferring the cells into culture media containing 0%, 0.1%, or 1% FCS. Cells were seeded into E-plate wells and monitored using the xCELLigence system for five days. **B** HOS, OS921 and CT-26 cells were stained with PKH26 and seeded to wells of 12-well plates and the next day half of the wells were transfered to 0.1% FCS (day “0”) Cells that had stopped proliferating remained red, while proliferating cells lost their color with each division. Then, cells of all three lines were removed from the substrate and the number of PKH26-positive cells in each sample was determined using flow cytophotometry (five samples for each time point. The experiment was repeated twice). **C** Cell cycle dynamics in HOS, OS921 and CT-26 cells following transfer to 0.1% FBS medium, with the deepest cell cycle arrest observed on day 5, defining the dormant state. **D** Expression of proliferation (Aurka, Ki67), **E** autophagy (ULK1, Atg7), **F** EMT (Zeb1, TWIST, Vimentin), and **G** stemness (CD44, ALDH1) markers in proliferating and dormant (day 5, 0.1% FBS) HOS, OS921 and CT-26 cells cells, assessed by RT-PCR. **H** Western blot of proliferating and dormant CT-26 cells (5 days in 0.1% FCS) with antibodies against autophagy markers. **I** HOS, OS921 and CT-26 c cells were seeded in 96-well plates. After cell attachment, 5.8 µM etoposide was added to osteosarcoma cells and 30 µM OxP for CT-26 cells, and cell viability was assessed via the MTT assay at 24 h. *p < 0.05; **p < 0.0001.

Conventional cancer treatments, such as chemotherapy and radiotherapy, often fail to completely eradicate all cancer cells, leading to the survival of residual populations and the enrichment of stem-like dormant cells. These cells exhibit enhanced resistance to therapy and increased autophagy activity, contributing to the formation of micrometastases and elevating the risk of cancer recurrence [35]. To investigate the mechanism by which CQ, in combination with OxP, dramatically suppresses metastasis, we established an in vitro dormancy model using serum depletion in cancer cells, and compared CQ’s effects on the viability of proliferating versus dormant cancer cells. When tumor cells acquire a dormant phenotype, they cease proliferating, as indicated by a decrease in the Cell Index upon culture in media containing 0%, 0.1%, or 1% FCS (Fig. 7A). This is further supported by the retention of PKH26 staining in the vast majority of dormant cells, in contrast to proliferating cells, where the proportion of stained cells progressively decreased in HOS, OS921, and CT-26 populations (Fig. 7B, Fig. S3). Dormant cells were also arrested in the G0/G1 phase of the cell cycle (Fig. 7C). By day 5 of serum deprivation, these cells showed reduced expression of proliferation markers Aurka and Ki67 (Fig. 7D), along with increased expression of autophagy-related genes ULK1 and Atg7 (Fig. 7E), as well as elevated levels of EMT markers ZEB1, TWIST, and Vimentin (Fig. 7F). Moreover, presumptive dormant cells acquired cancer stem cell–like characteristics, including upregulation of CD44 and ALDH1 (Fig. 7G). In CT-26 cells, dormancy was associated with increased expression of autophagy markers Atg7 and LC3-II, along with decreased levels of p62 (Fig. 7H). Dormant cells also showed resistance to antitumor drugs, Eto and OxP (Fig. 7I).

**Fig. 8:**
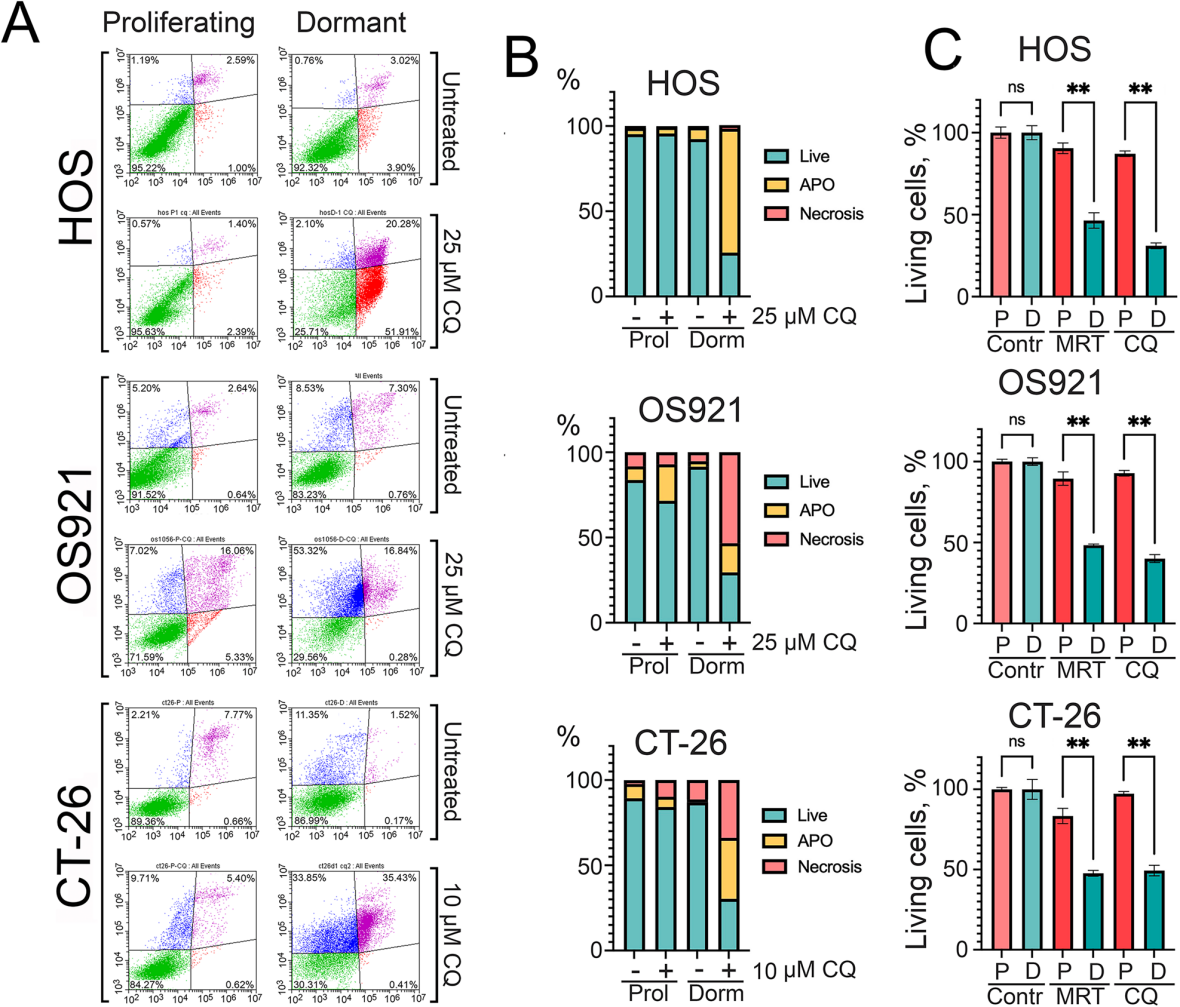
Chloroquine exhibits greater toxicity toward dormant cancer cells than proliferating cells. **A** HOS, OS921 and CT-26 cells were transferred to 0.1% FCS and five days later dormant and proliferating cells were treated with 25 µM CQ for osteosarcoma cells and with 10 µM CQ and after 24 h were stained with Annexin V/PI and subjected for flow cytometry. Flow cytometry plots of proliferating and dormant HOS, OS921 and CT-26 cells treated with CQ. **B** Quantification of viable, apoptotic, and necrotic cells, calculated from three independent experiments. **C** HOS, OS921 and CT-26 cells were seeded to wells of 96-well plate. Then cells were treated with CQ as in “A” and with 3 µM MRT68921. Test MTT was performed after 24 h of incubation. *p < 0.05; **p < 0.0001.

Importantly, dormant HOS, OS921, and CT-26 cells also exhibited another key feature of cancer stem cells: enhanced resistance to therapy. Using Annexin V/PI staining and flow cytometry, we found that chloroquine (CQ) selectively induced cell death in the dormant population, primarily via necrosis, although apoptosis also contributed (Fig. 8). After CQ treatment, 95.6% of proliferating HOS cells remained viable, compared to only 25.7% of dormant cells. Similarly, 71.6% of proliferating OS921 cells survived versus 29.6% of their dormant counterparts. In CT-26 cells, 84.3% of proliferating cells survived CQ treatment, compared to only 30.3% of dormant cells (Fig. 8A, B). MTT assay results were consistent with Annexin V staining. In the same experiment, we also tested 3 μM MRT68921, and found that both autophagy inhibitors predominantly induced death in the dormant cell population (Fig. 8C).

These findings suggest that in our in vivo experiments (Fig. 6), the combination of OxP and CQ targeted distinct tumor cell populations, whereby OxP primarily eliminated proliferating cancer cells, while CQ, primarily by suppressing autophagy, effectively eradicated resistant dormant cells that aroused as a result of chemotherapy. This dual mechanism likely contributes to the significant reduction in metastasis observed in the CQ + OxP treatment group.

## Discussion

Resistance to anticancer drugs, tumor relapse, and metastasis remain major challenges in cancer therapy, with extensive research has been dedicated to address these challenges in cancer patients. Among the various strategies explored, combination therapies have emerged as a promising approach to circumvent resistance mechanisms by integrating conventional chemotherapies with immunotherapies, targeted therapies, and inhibitors of cellular proteostasis, beyond others. Our research group has focused on disrupting tumor cell resistance by targeting key components of the cellular proteostasis system. For instance, we previously demonstrated that the HSF1 inhibitor CL-43 substantially sensitized colorectal and lung carcinoma cells to chemotherapy [36, 37]. Here, we explored autophagy inhibition as a potential strategy to counteract therapy resistance.

While the role of autophagy in cancer is highly context-dependent and varies across tumor types, accumulating evidence underscores its critical involvement in cancer progression and therapy resistance [16, 38]. Autophagy is induced in response to tumor-associated hypoxia and has been shown to mediate chemoresistance in colon cancer models [39, 40]. Furthermore, several studies have reported a protective role of autophagy in osteosarcoma, glioblastoma, and acute myeloid leukemia, where its inhibition using CQ significantly reversed chemoresistance [41, 42]. Recent findings, including our own, suggest that autophagy serves as a pro-survival compensatory mechanism following cellular proteostasis disruption, such as Hsp70 inhibition or proteasome blockade. However, this adaptive response can be effectively counteracted using autophagy inhibitors like CQ and SAR405 [43–48]. Thus, autophagy likely emerges as a universal hallmark of chemoresistance, enabling cancer cells to withstand therapy-induced stress.

In our work, we examined not only established osteosarcoma cell lines but also patient-derived lung metastasis cells from individuals with osteosarcoma who had undergone multiple courses of therapy and experienced recurrent relapses. These patient-derived cells exhibited pronounced resistance to etoposide and showed enhanced autophagy activation in response to treatment. This observation led us to explore the therapeutic potential of autophagy inhibition by combining CQ with standard anticancer drugs. Our findings demonstrate that pre-treatment with CQ, administered 16 h prior to etoposide, significantly impaired osteosarcoma cell resistance and sensitized them to chemotherapy. This was confirmed using multiple experimental approaches, including MTT assays, apoptosis quantification, xCELLigence real-time monitoring, and 3D spheroid models.

CQ has been widely studied as a monotherapy and has been shown to induce cell death across various cancer types [27, 49–51]. Preclinical studies have further demonstrated that CQ inhibits autophagy and reduces tumor growth in bladder cancer and pancreatic adenocarcinoma models [52]. Additionally, the combination of CQ with alkylating agents has been shown to suppress tumor growth in a mouse model of B-cell lymphoma [53, 54].

Metastasis remains a key challenge in cancer therapy, often limiting curative treatment options [55]. In our study, the combination of CQ with anticancer drugs significantly downregulated EMT markers and reduced cell motility in both osteosarcoma and CT-26luc colorectal cancer cells. In colony formation assays, CQ-treated osteosarcoma cells lost their ability to resume tumor growth, highlighting its potential in preventing cancer cell repopulation and recurrence. Furthermore, half of the treated animals with osteosarcoma, the primary tumor failed to reach a sufficient size for analysis, underscoring the potent efficacy of this therapeutic approach.

Although numerous preclinical studies have investigated CQ as an autophagy inhibitor in combination with chemotherapy, most in vivo experiments have relied on subcutaneous tumor models [39, 50–56]. However, these models do not accurately recapitulate the tumor microenvironment and fail to mimic the metastatic process. In contrast, our study employed an orthotopic model in which CT-26luc cells were injected into the submucosa of the mouse cecum. This approach closely resembles the natural progression of colorectal cancer in patients and facilitates spontaneous metastasis to distant organs, such as the liver, lungs, and spleen. As a result, we observed metastases in the liver, lungs, and spleen in untreated animals and in those receiving monotherapy. Importantly, no detectable metastases were found in mice treated with the OxP + CQ combination, as confirmed by the absence of luciferase signals in metastatic organs.

Our findings further indicate that CQ may substantially suppress metastasis by primarily targeting dormant cancer cells. In our in vitro dormancy model, CQ was significantly more cytotoxic to dormant cells than proliferative ones. This suggests that the combination of chemotherapy and CQ exerts a dual therapeutic effect: while conventional anticancer drugs eliminate actively proliferating tumor cells, CQ effectively eradicates dormant cells, which are otherwise resistant to standard therapies [16, 57]. This therapeutic approach may provide a more comprehensive strategy for preventing both tumor regrowth and metastasis.

Our study demonstrates that autophagy inhibition via CQ enhances the therapeutic efficacy of etoposide and oxaliplatin, leading to a significant reduction in cancer progression and metastasis compared to monotherapy. These findings, in conjunction with ongoing research efforts, support the potential of combining autophagy inhibitors with standard chemotherapy as a promising strategy for the treatment of osteosarcoma and colorectal cancer. Further investigations, particularly in clinical settings, are warranted to validate the translational relevance of this approach.

## Supplementary information

Wet blots

Supplementary Material

## Data Availability

All data obtained during this study are included in this published article and its supplementary materials.
