# Correction: Chloroquine overcomes chemotherapy resistance and suppresses cancer metastasis by eradicating dormant cancer cells

**DOI:** 10.1038/s41419-026-08552-0

**Published:** 2026-04-17

**Authors:** Marina A. Mikeladze, Liubov S. Kuznetcova, Elena Y. Komarova, Margarita A. Galcheva, Vladimir F. Lazarev, Lev A. Khamaev, Maria A. Konanova, Yana A. Gladova, Anna B. Danilova, Boris A. Margulis, Bashar A. Alhasan, Irina V. Guzhova

**Affiliations:** 1 Institute of Cytology of Russian Academy of Sciences, Tikhoretsky prospect, 4, S.Petersburg, Russia; 2 N.N.Petrov Scientific Medical Research Center of Oncology, Leningradskaya str, 68, Pesochny, S.Petersburg, Russia

**Keywords:** Cell death, Autophagy

Correction to: *Cell Death & Disease* 10.1038/s41419-025-08304-6, published online 10 December 2025

During an internal final review of the article, we identified an unintended duplication within one of the blot areas in Figure 1; specifically, the ATG5 blot in Figure 1G and 1H was inadvertently duplicated during figure assembly.

Incorrect Figure 1
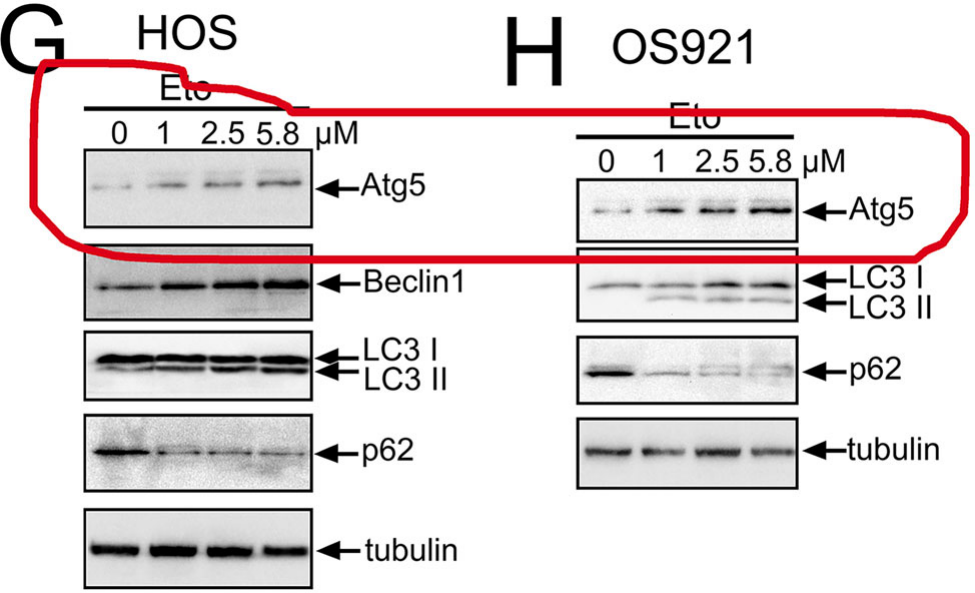


Correct Figure 1
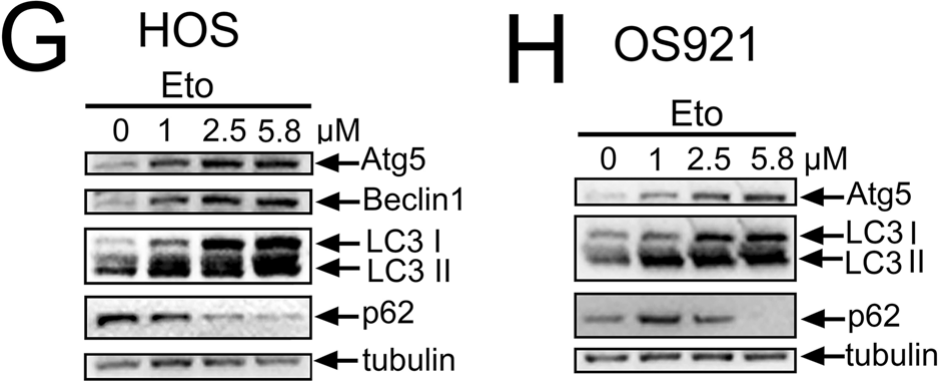


## Supplementary information

Wet blots for erratum

