# Supplementary material for: Chloroquine Overcomes Chemotherapy Resistance and Suppresses Cancer Metastasis by Eradicating Dormant Cancer Cells
Source: Cell Death Dis. 2025 Dec 10;17(1):91. doi: 10.1038/s41419-025-08304-6 (PMC12830959; doi:10.1038/s41419-025-08304-6)

Figure 1G (HOS cells):

Atg5:

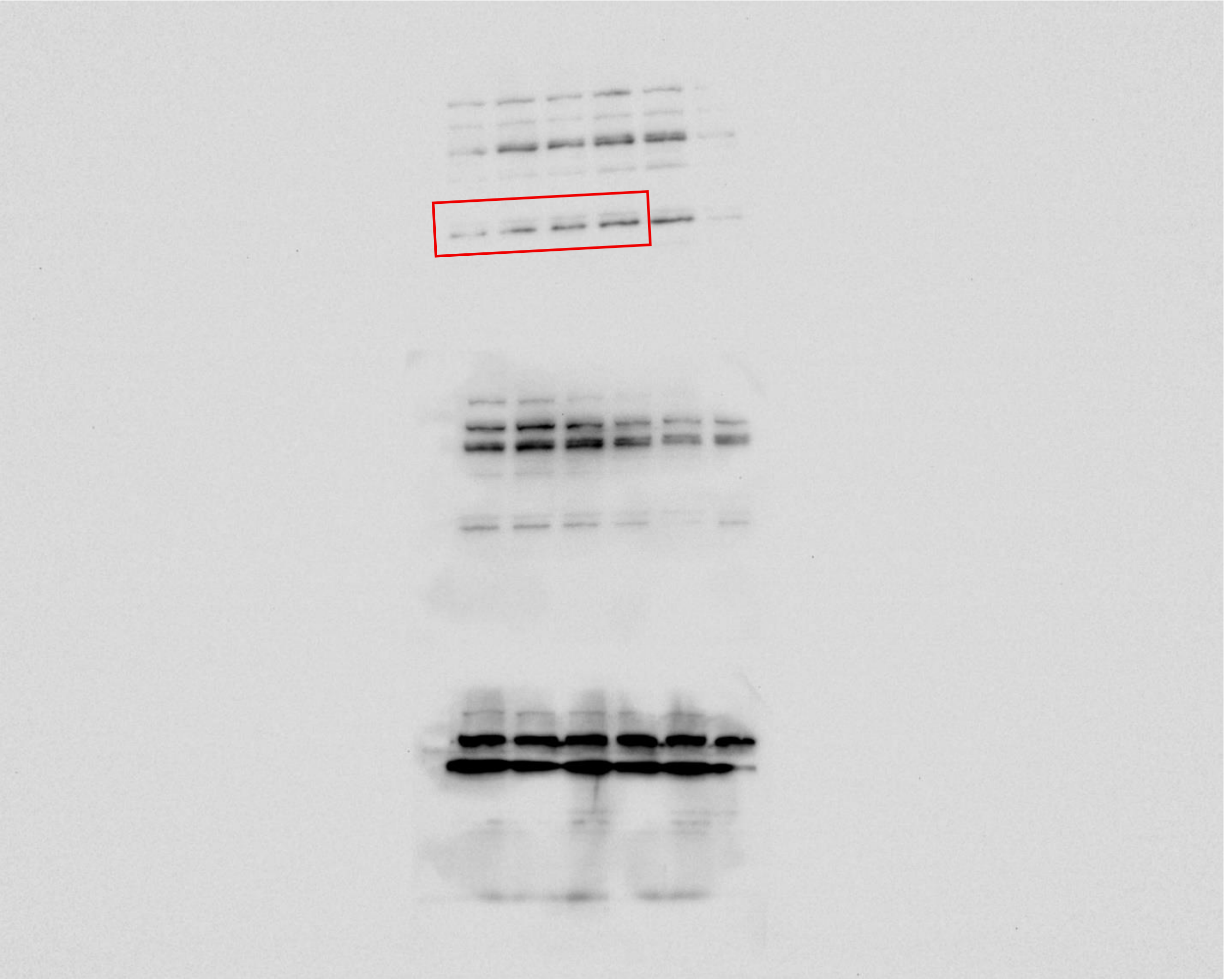

Figure 1G (HOS cells):

Beclin 1:

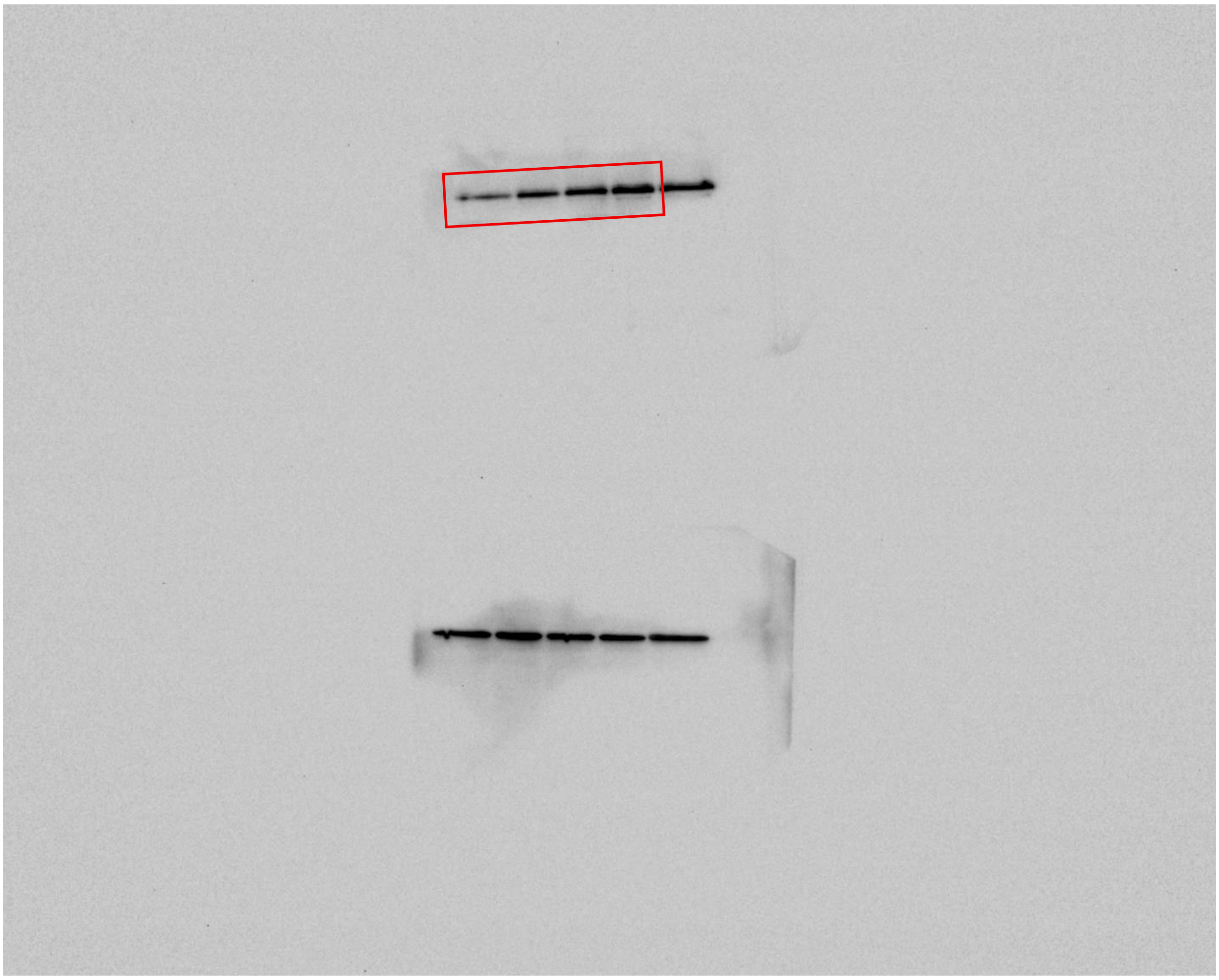

Figure 1G (HOS cells):

LC3 I/LC3 II:

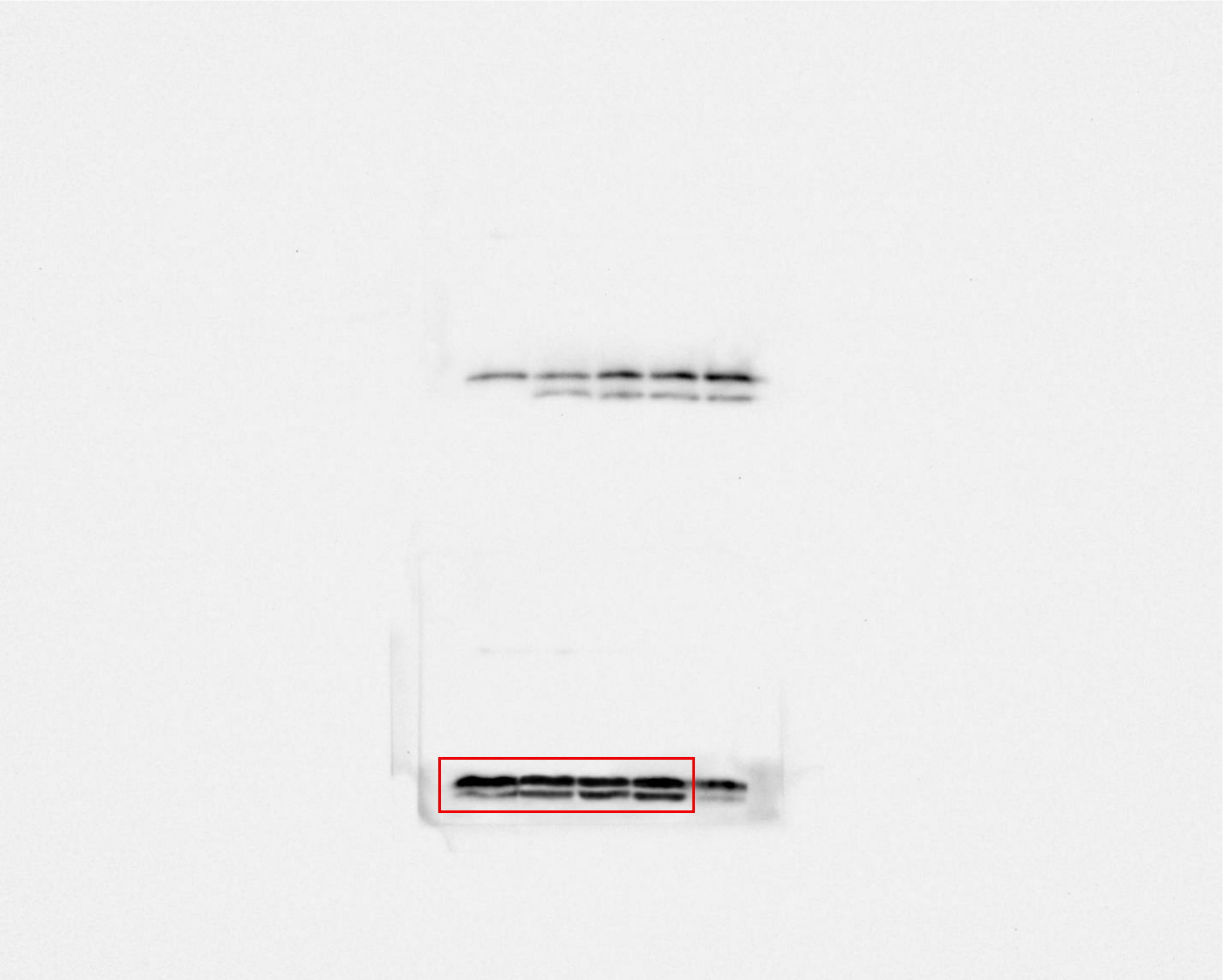

Figure 1G (HOS cells):

P62:

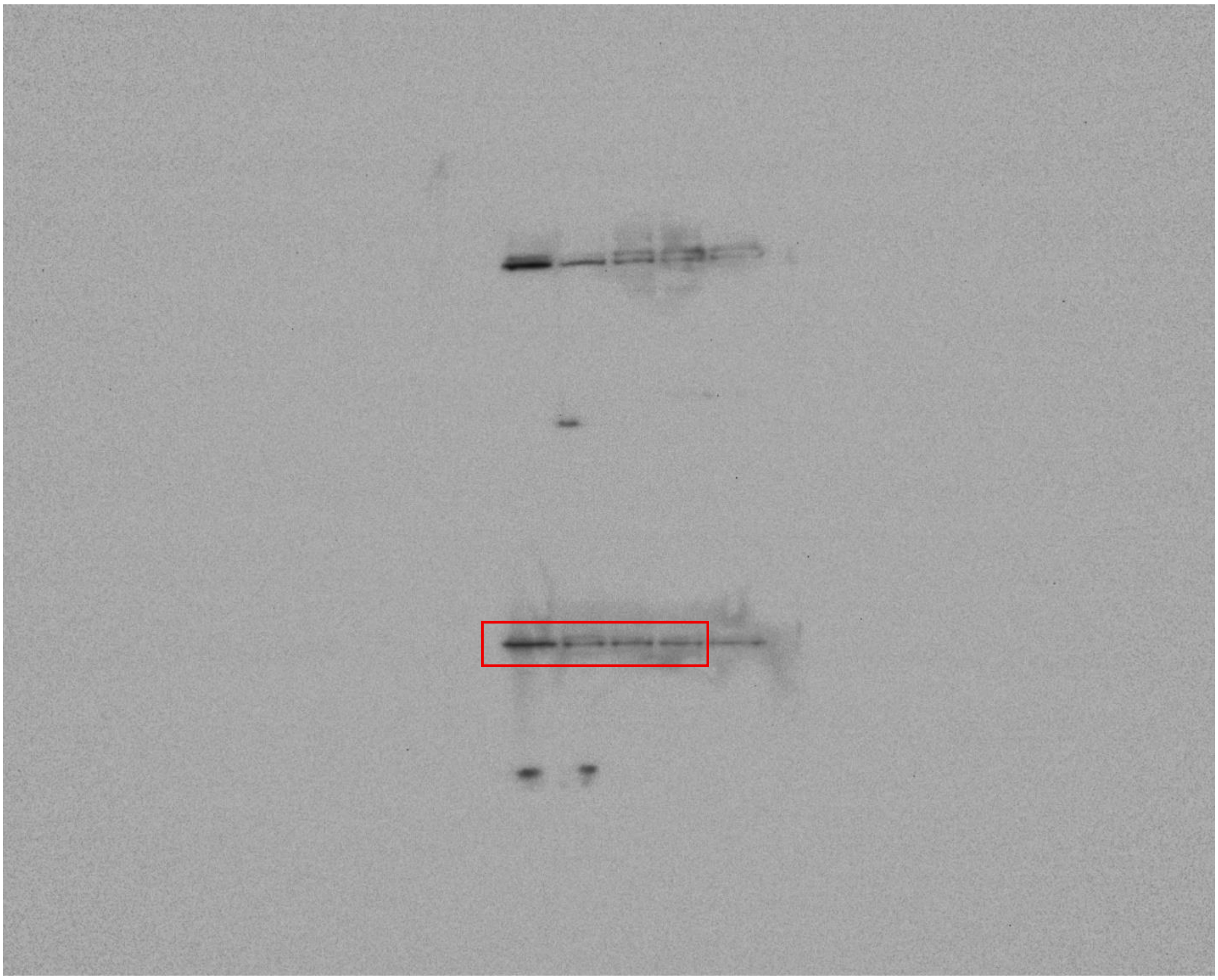

Figure 1G (HOS cells):

tubulin:

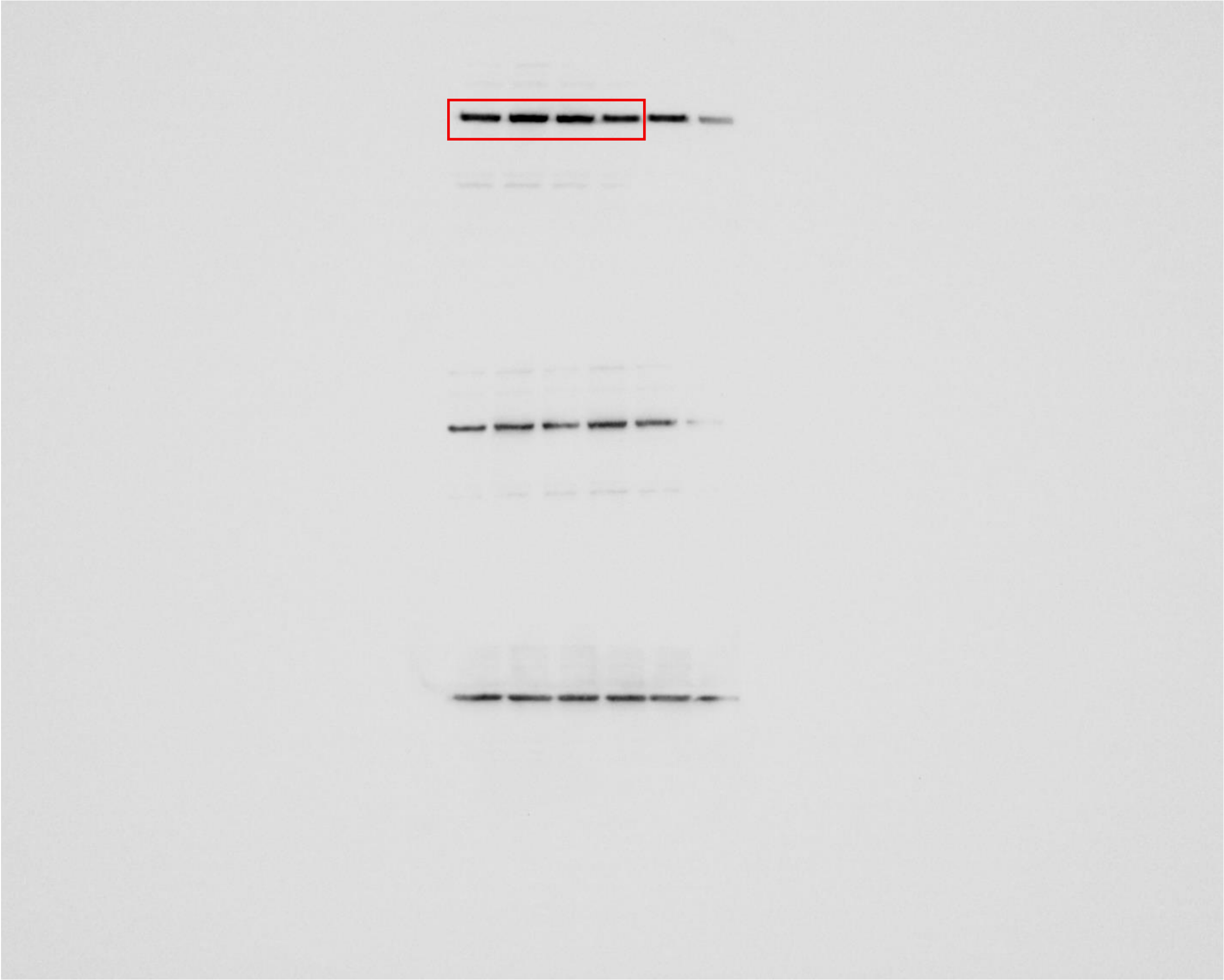

Figure 1H (OS921 cells):

Atg5:

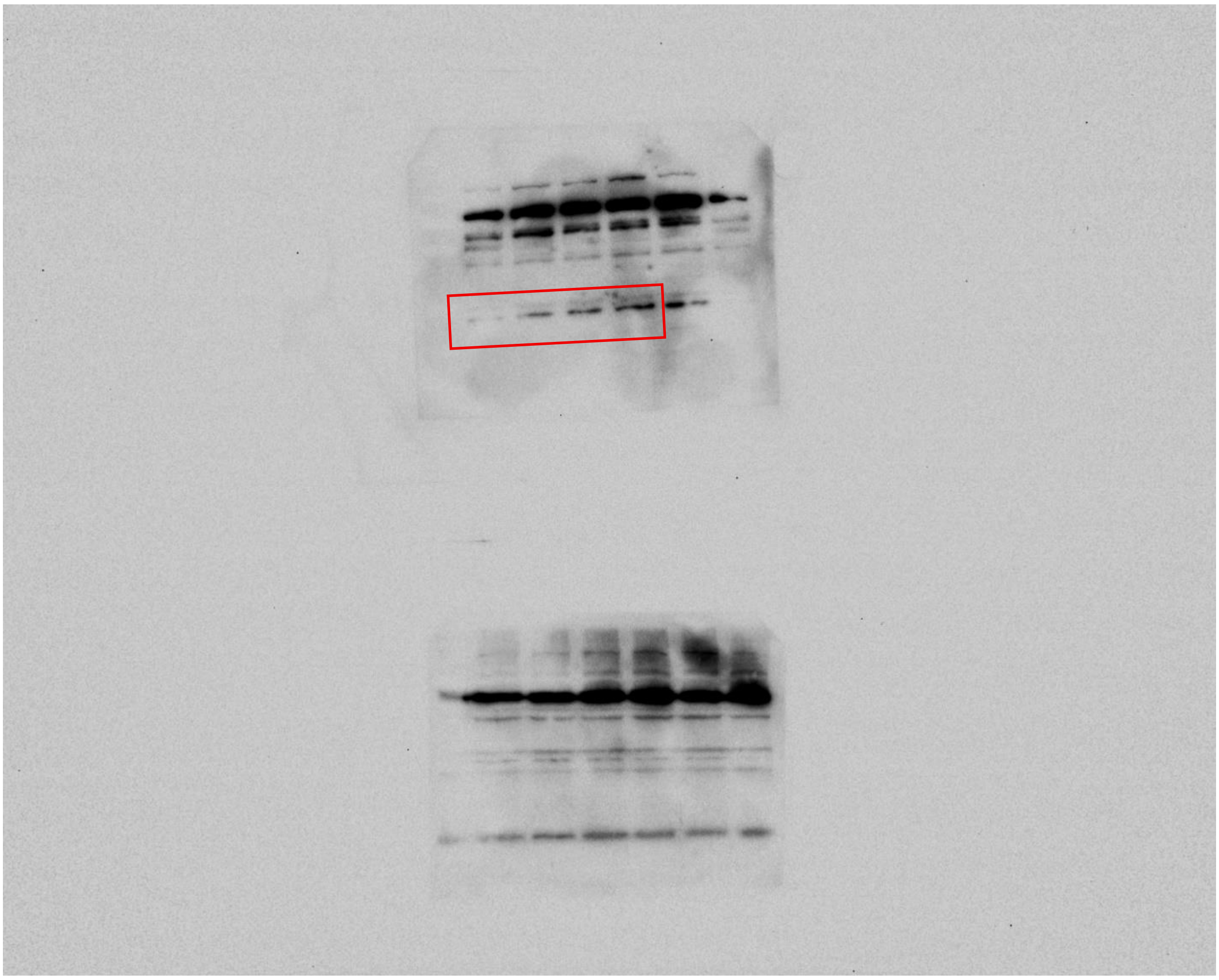

Figure 1H (OS921 cells):

LC3 I/LC3 II:

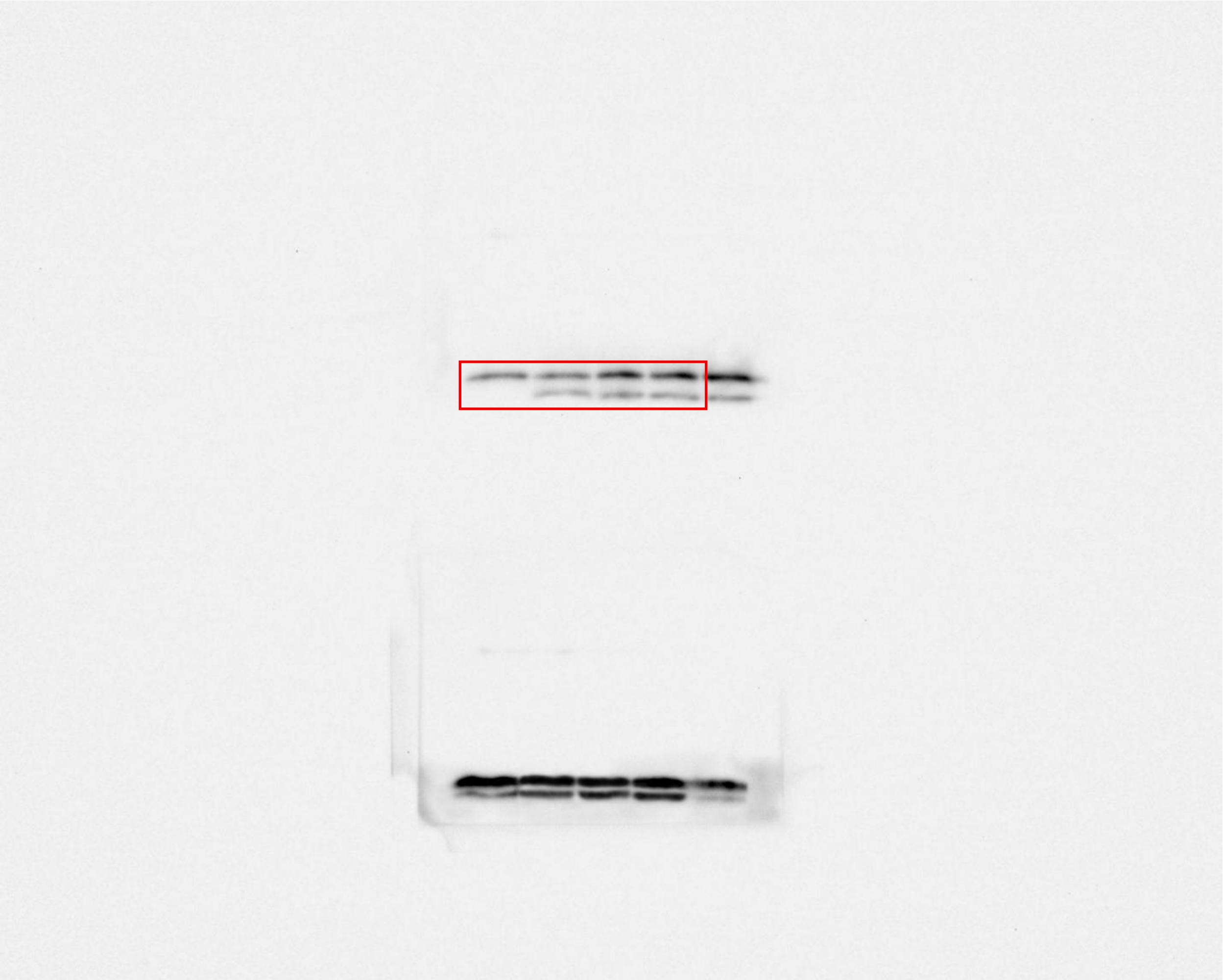

Figure 1H (OS921 cells):

P62:

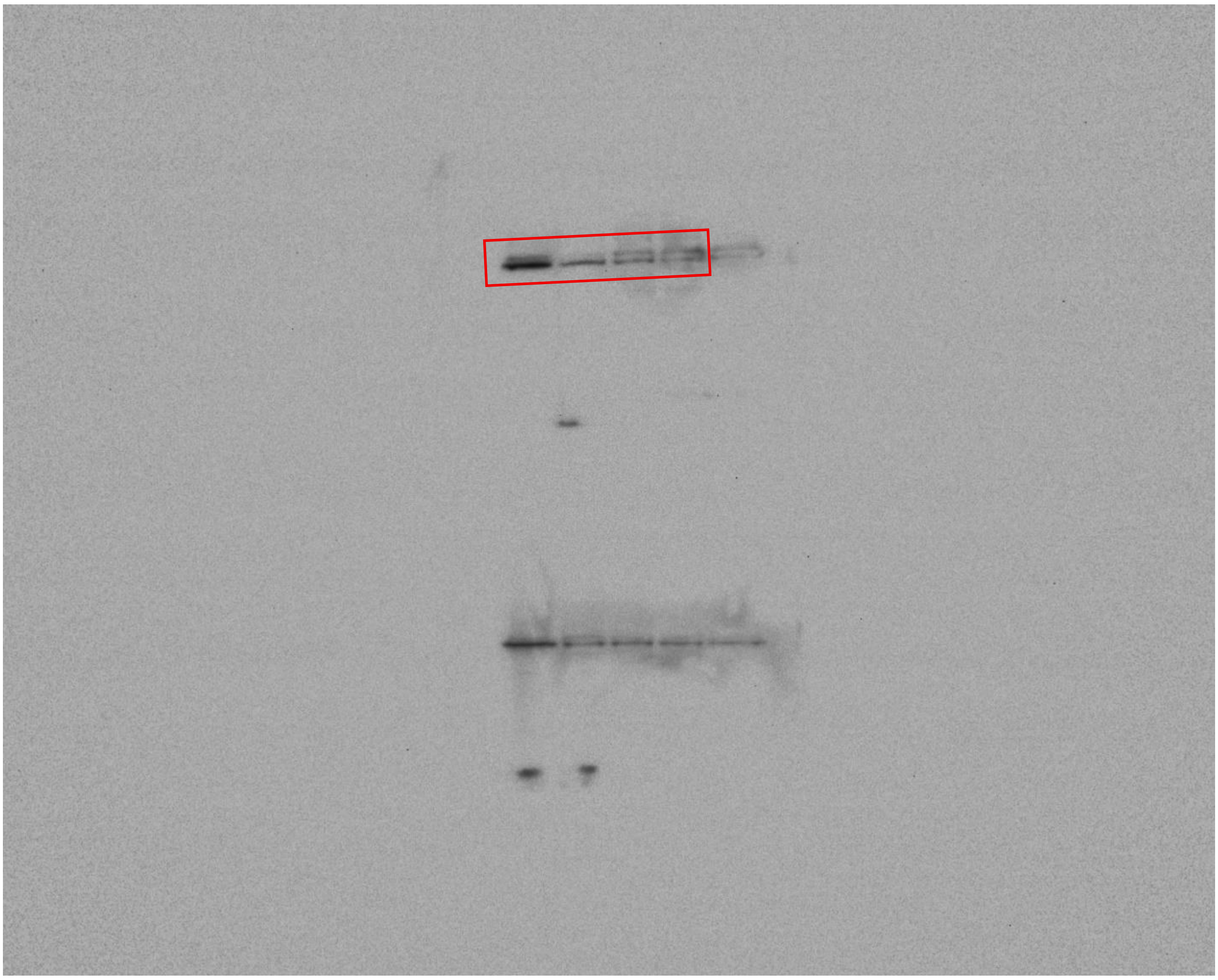

Figure 1H (OS921 cells):

tubulin:

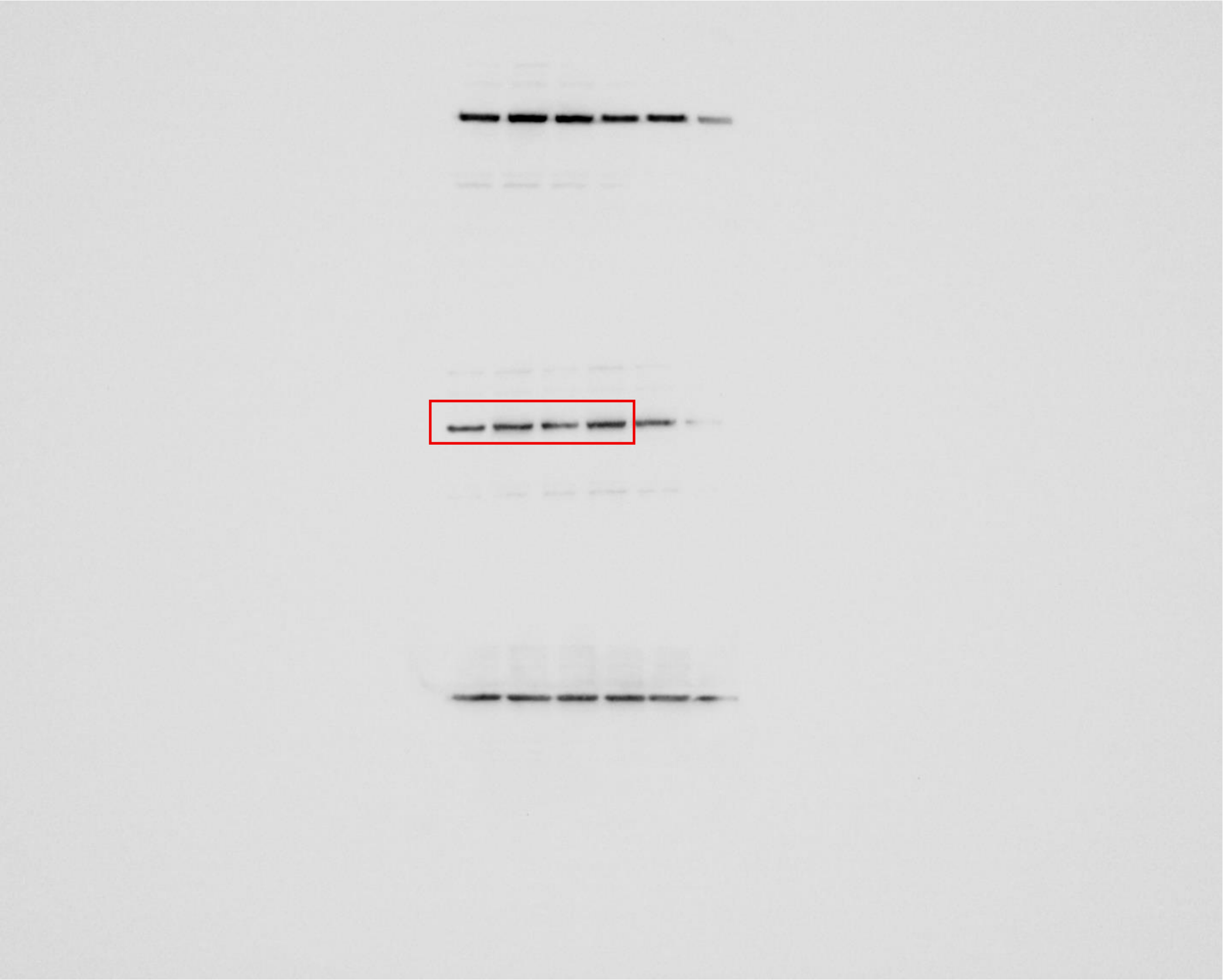

Figure 1I (OS1056 cells):

Atg5:

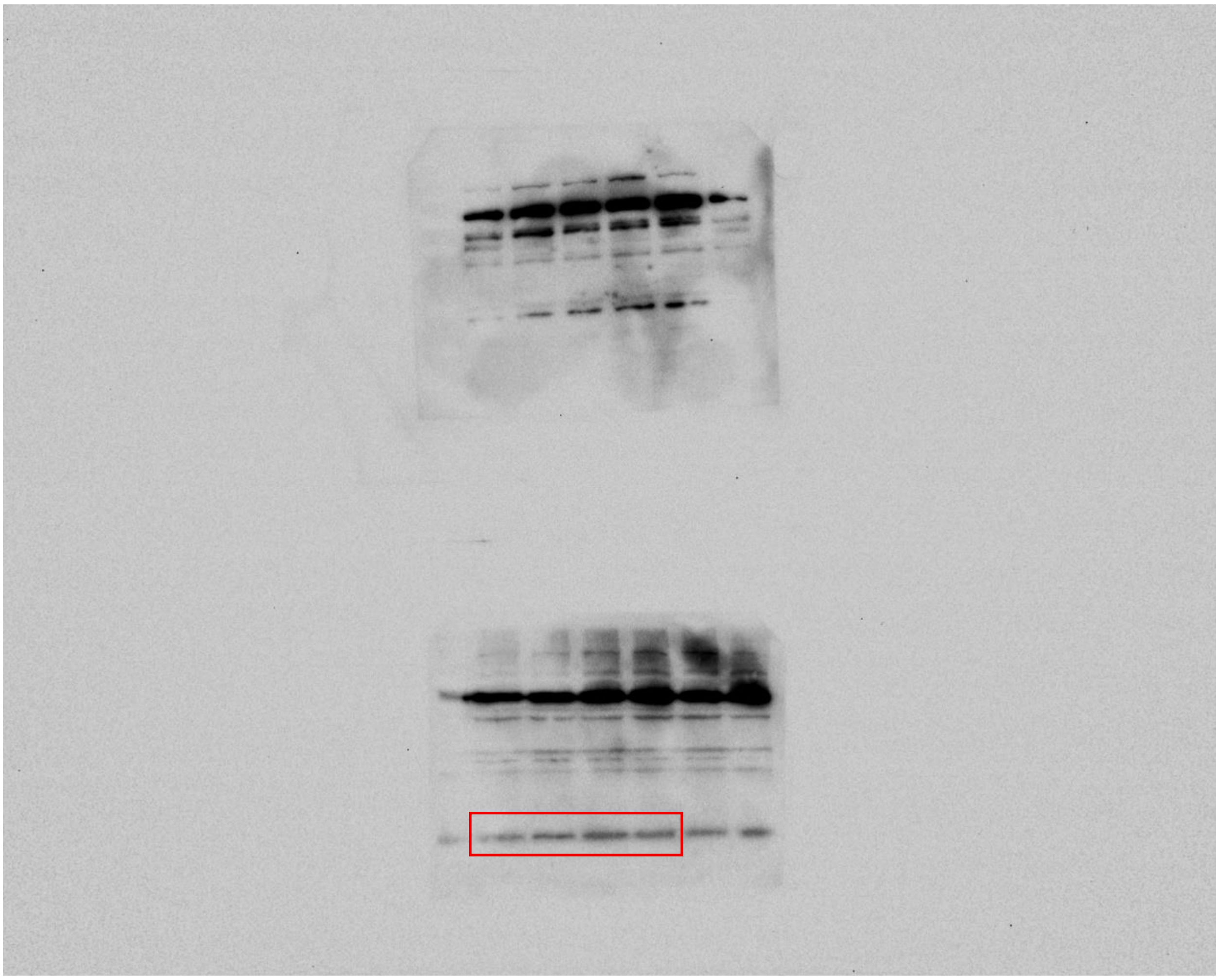

Figure 1I (OS1056 cells):

LC3 I/LC3 II:

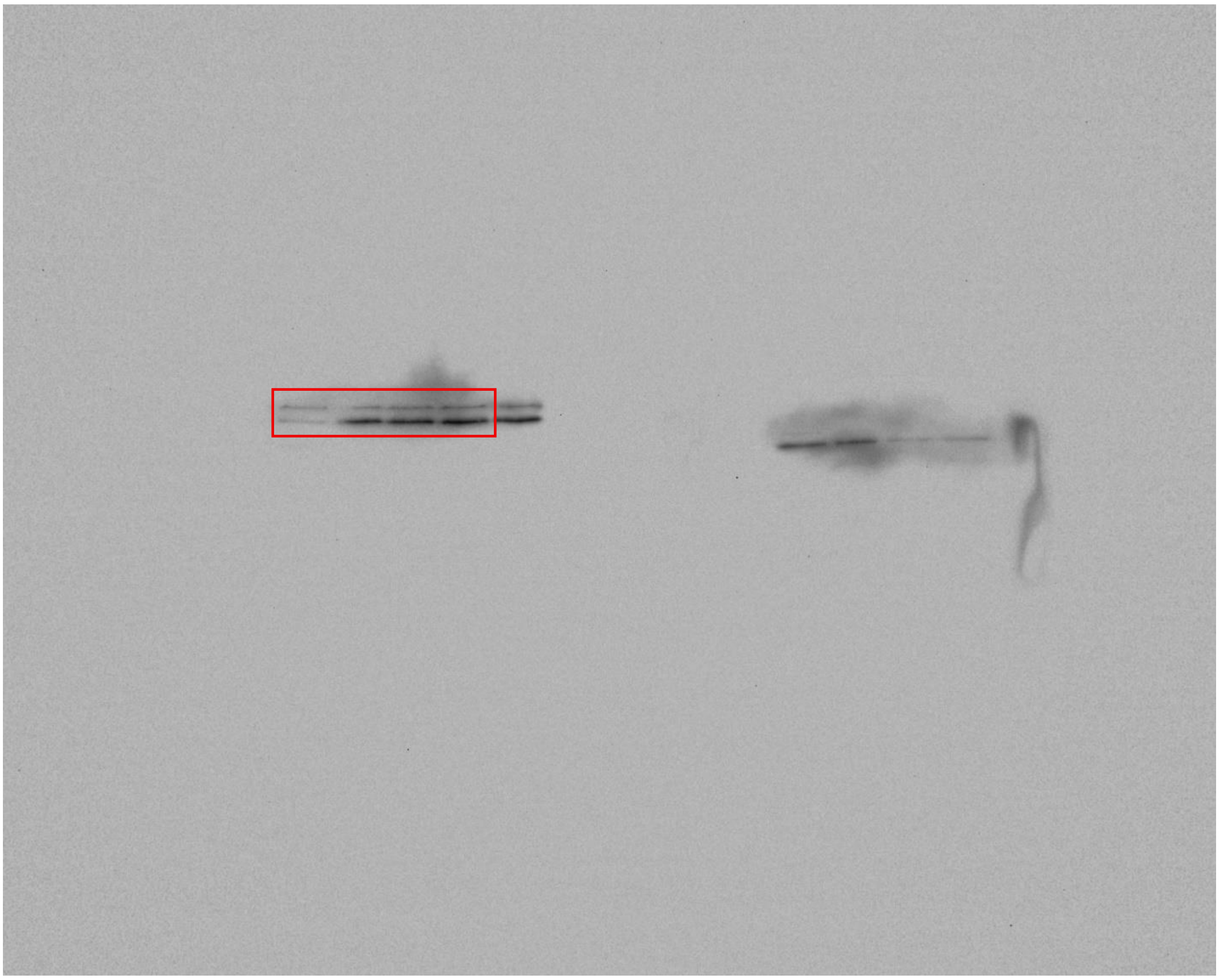

Figure 1I (OS1056 cells):

P62:

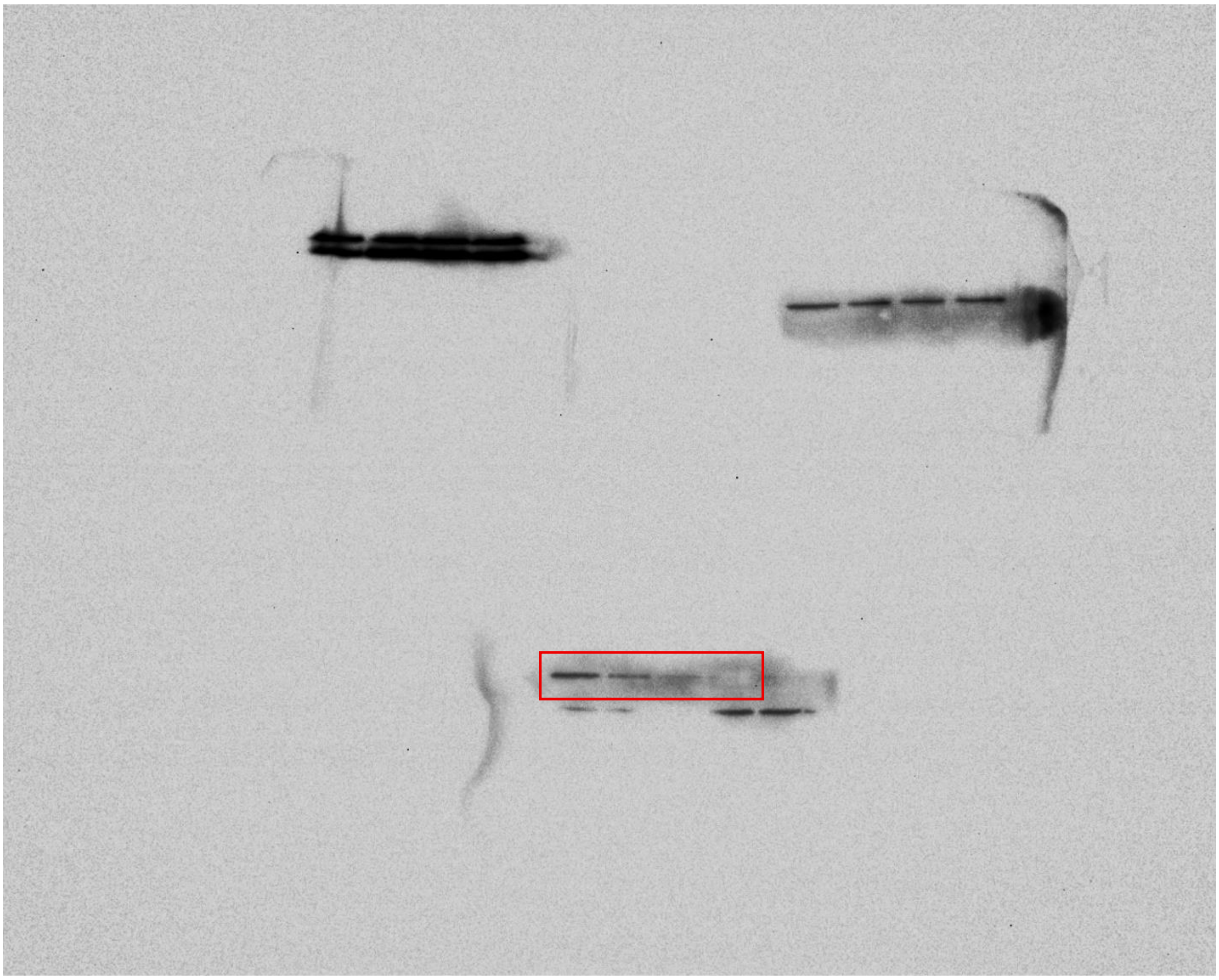

Figure 1I (OS1056 cells):

tubulin:

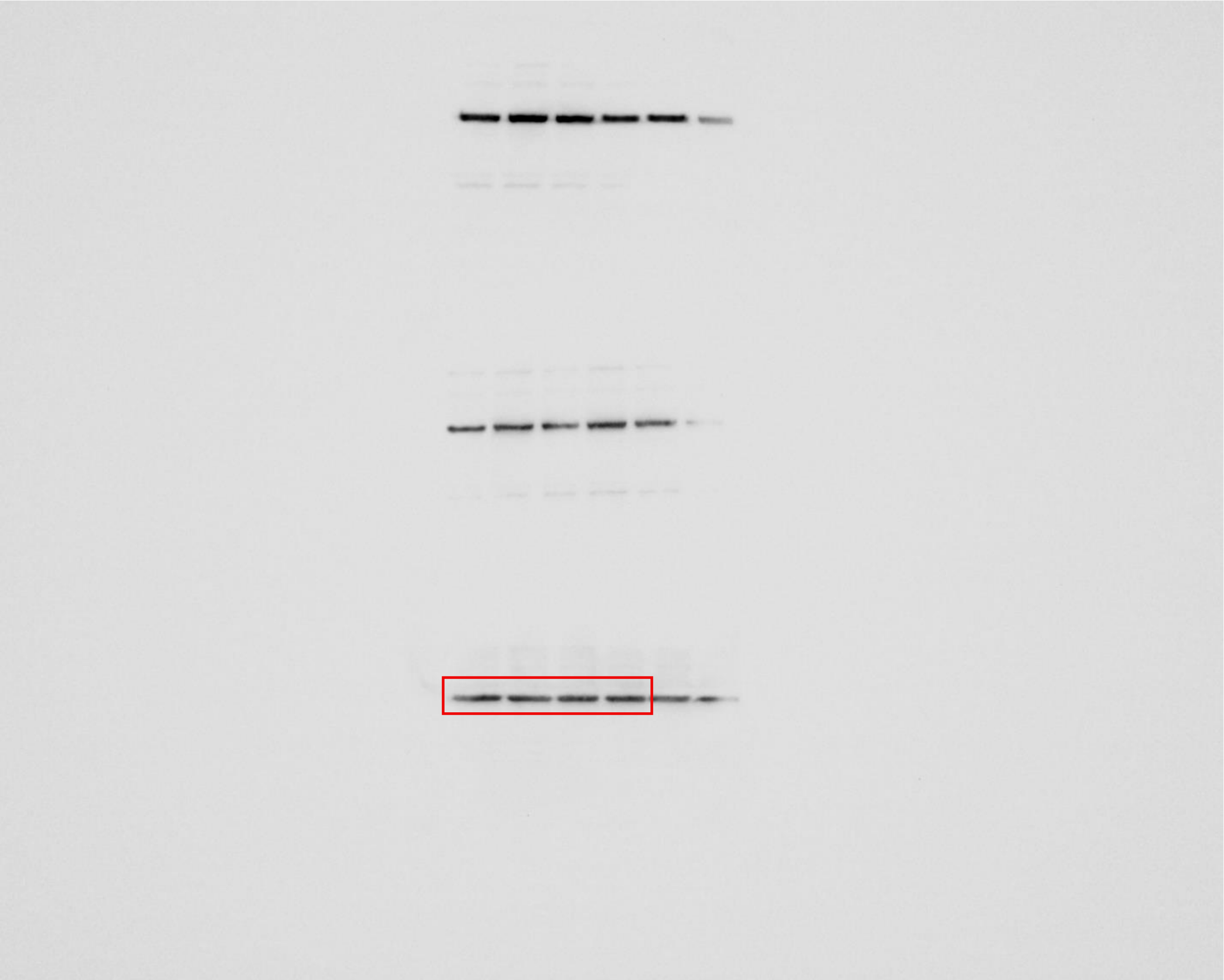

Figure 1J (CT-26 cells):

LC3 I/LC3 II:

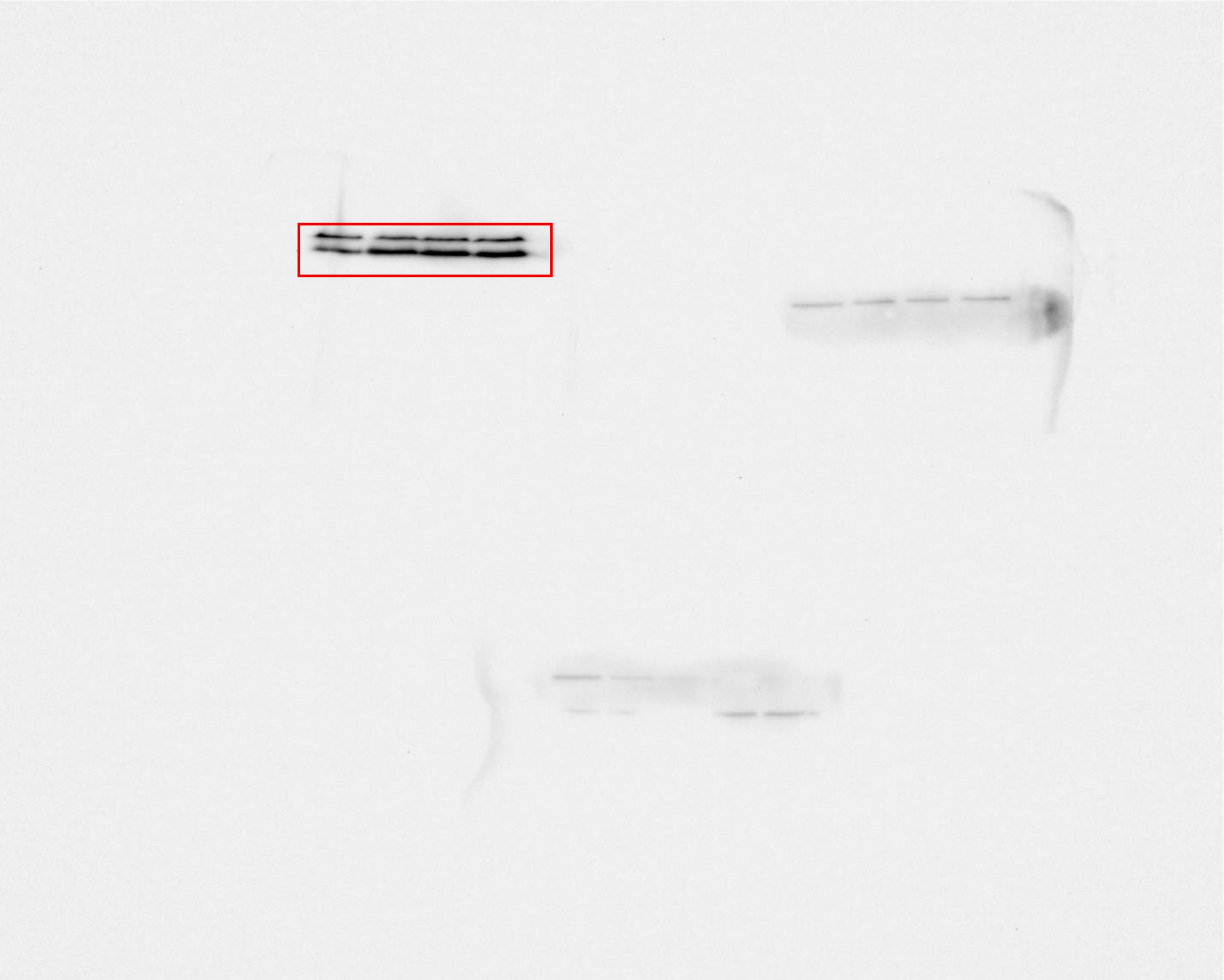

Figure 1J (CT-26 cells):

P62:

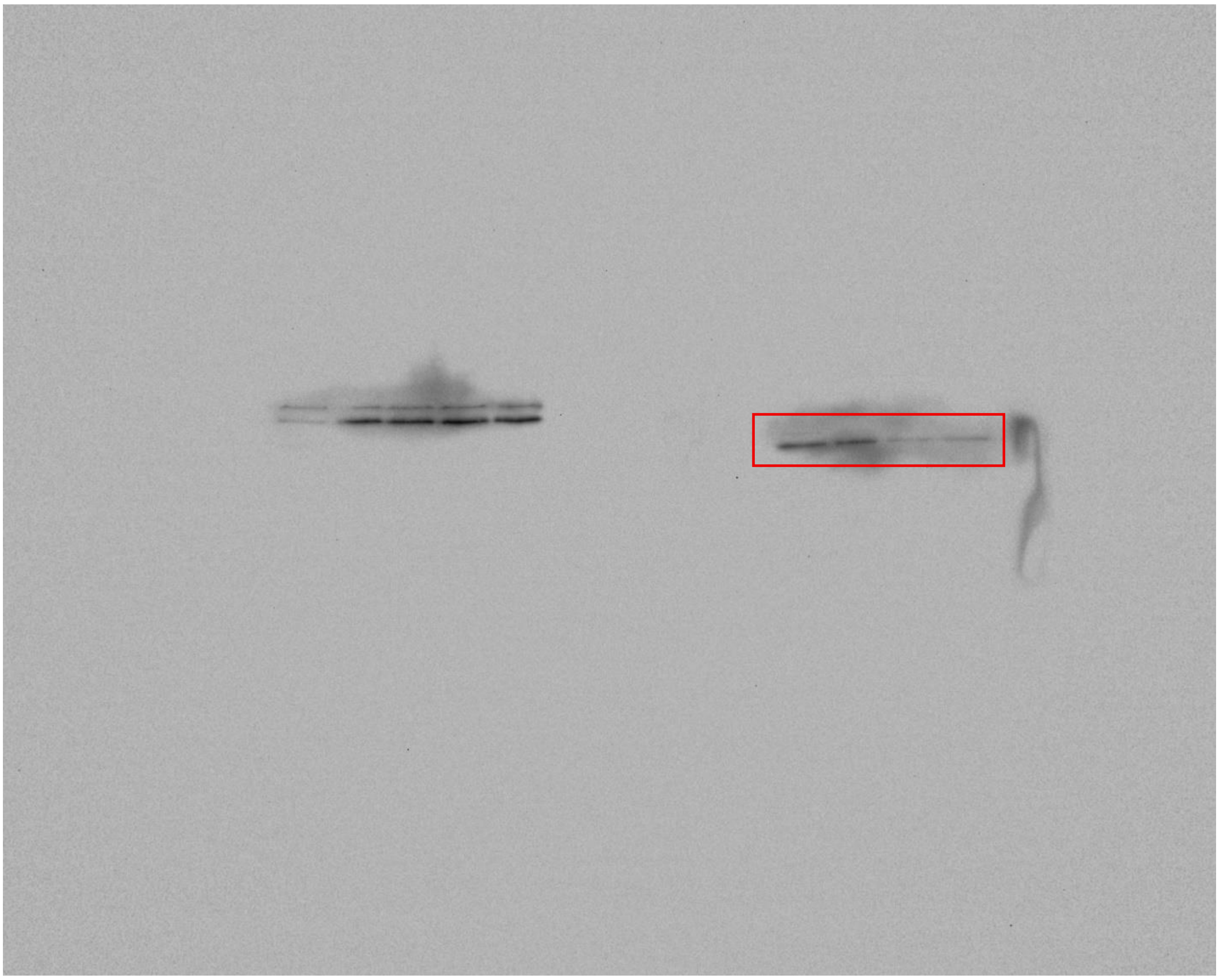

Figure 1J (CT-26 cells):

tubulin:

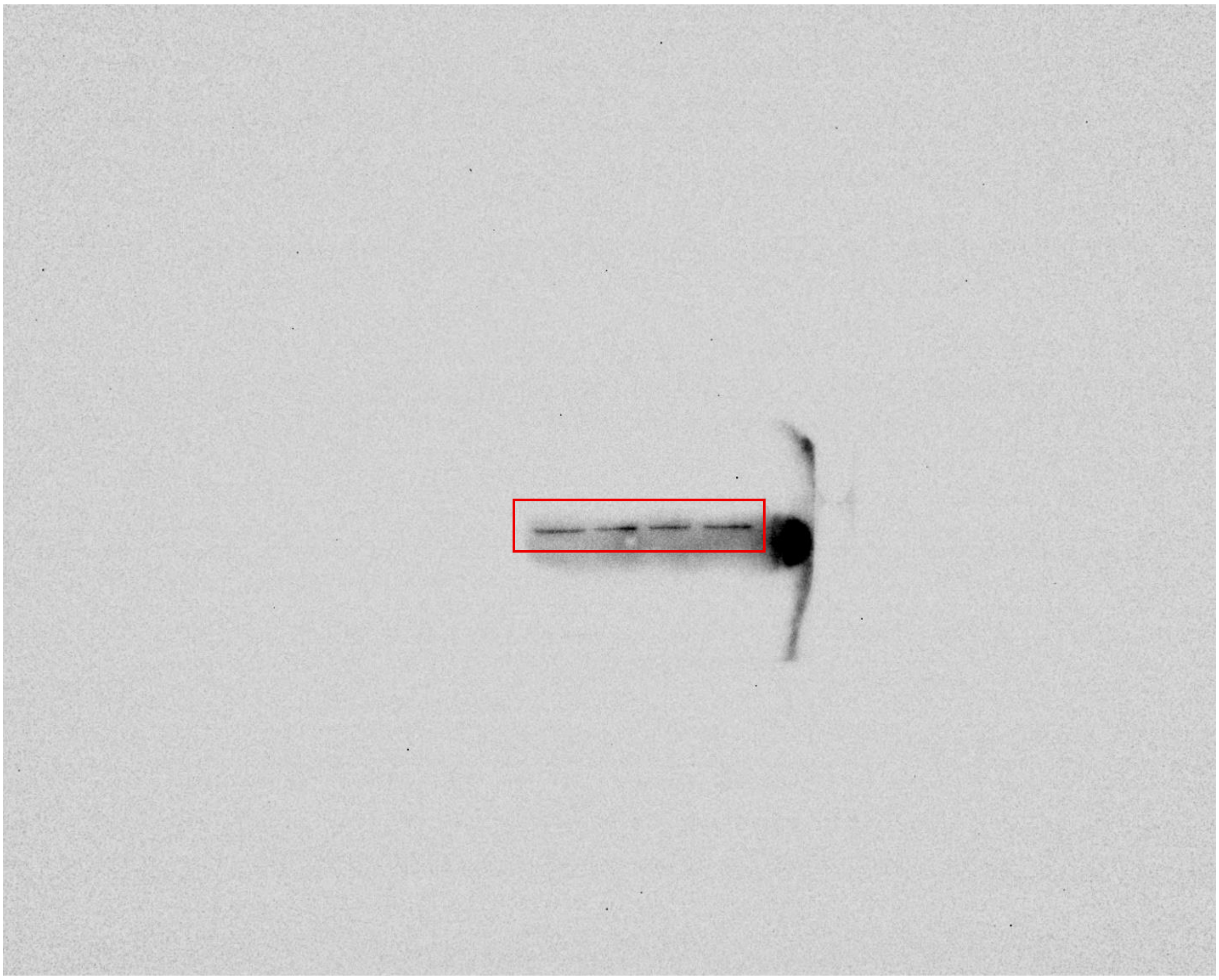

Figure 2B (OS921 cells):

Atg7:

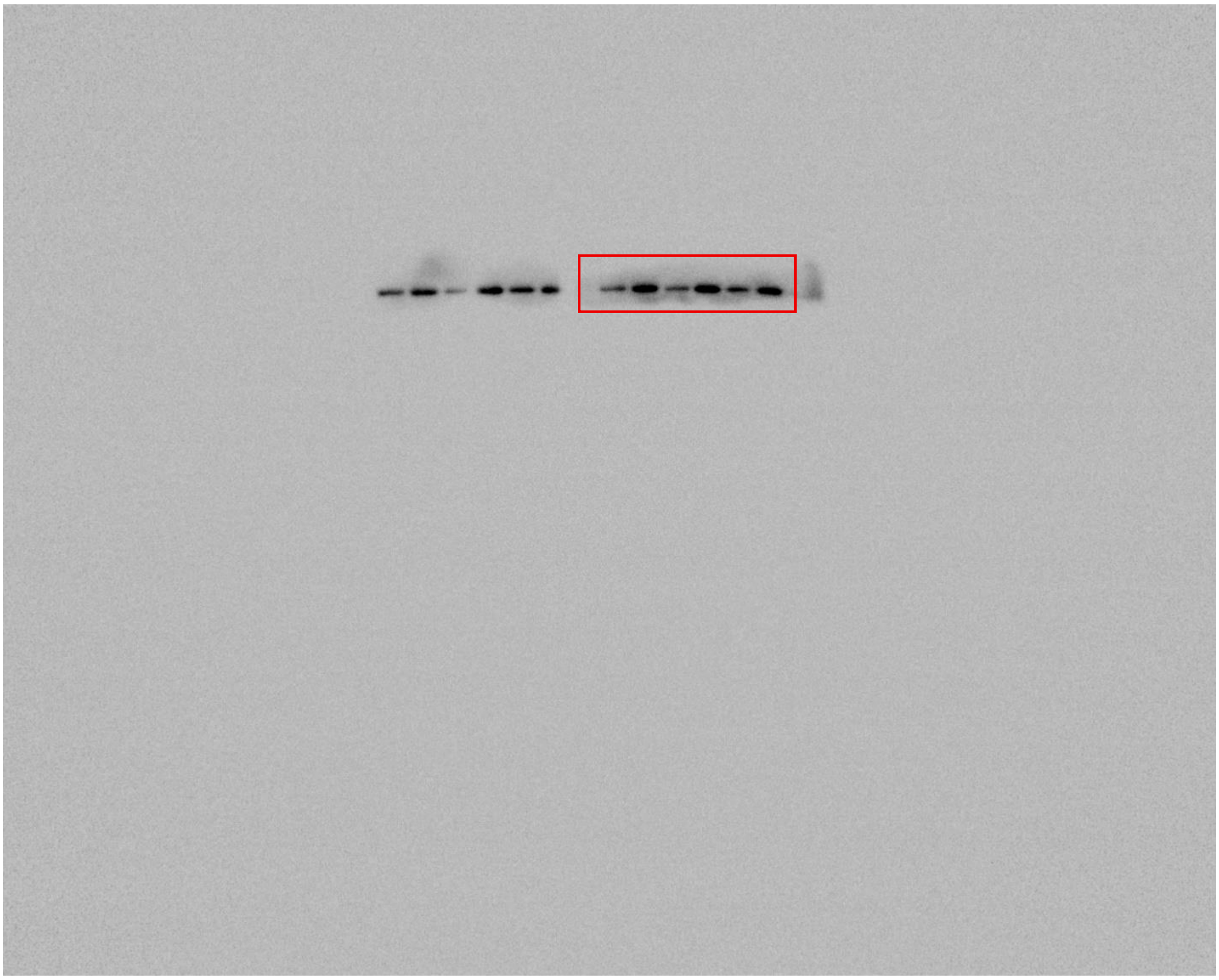

Figure 2B (OS921 cells):

tubulin:

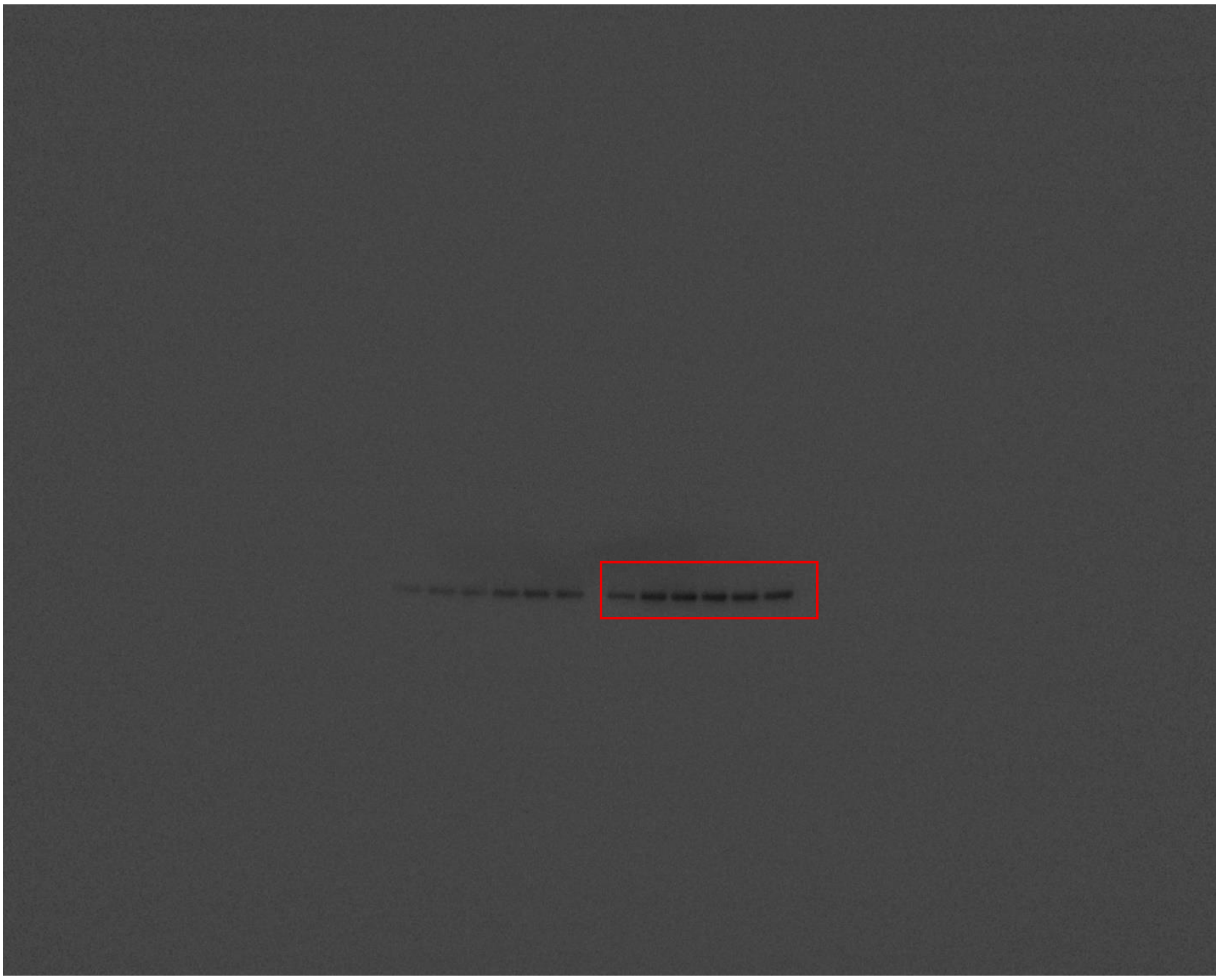

Figure 2B (OS921 cells):

P62:

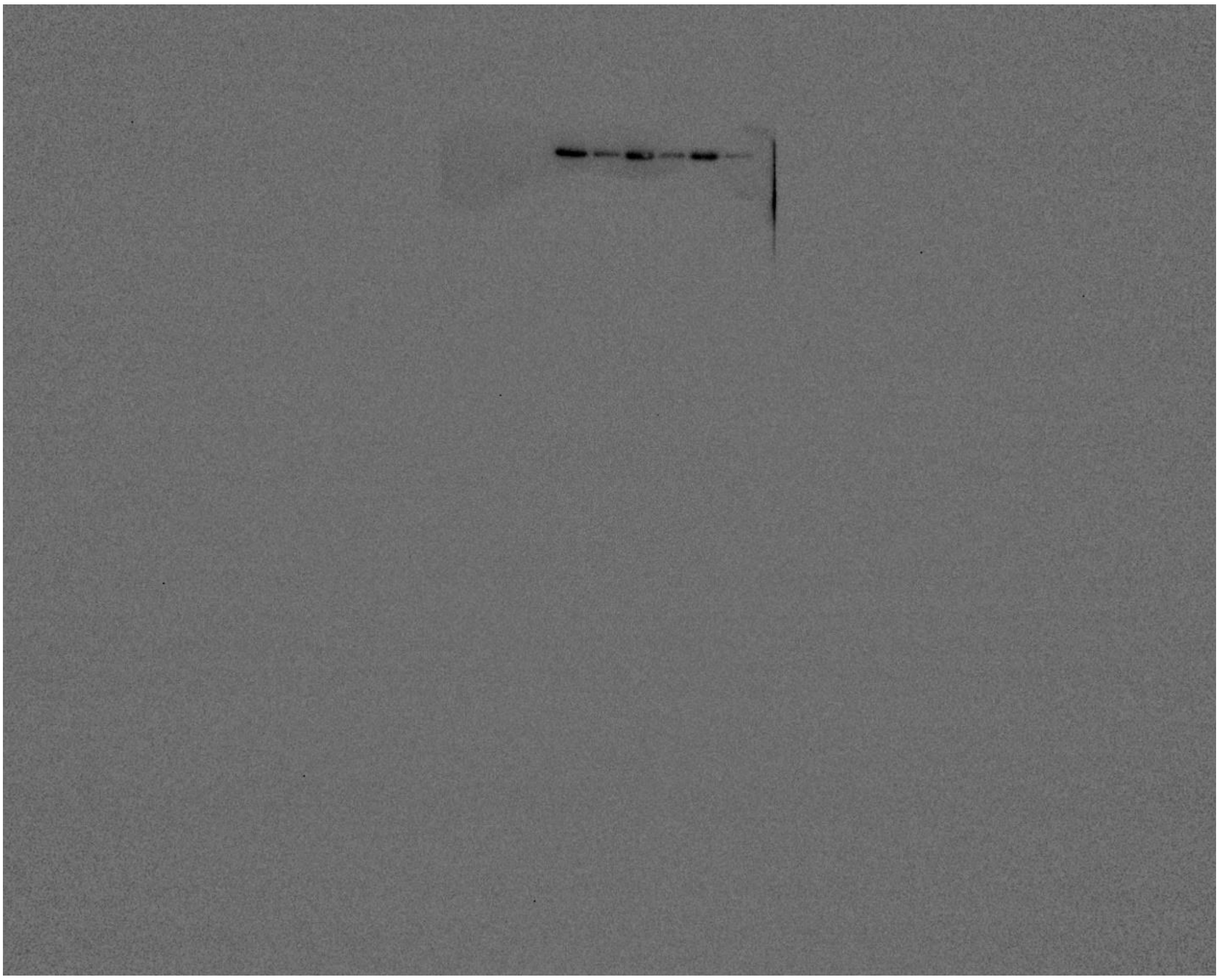

Figure 2B (OS921 cells):

tubulin:

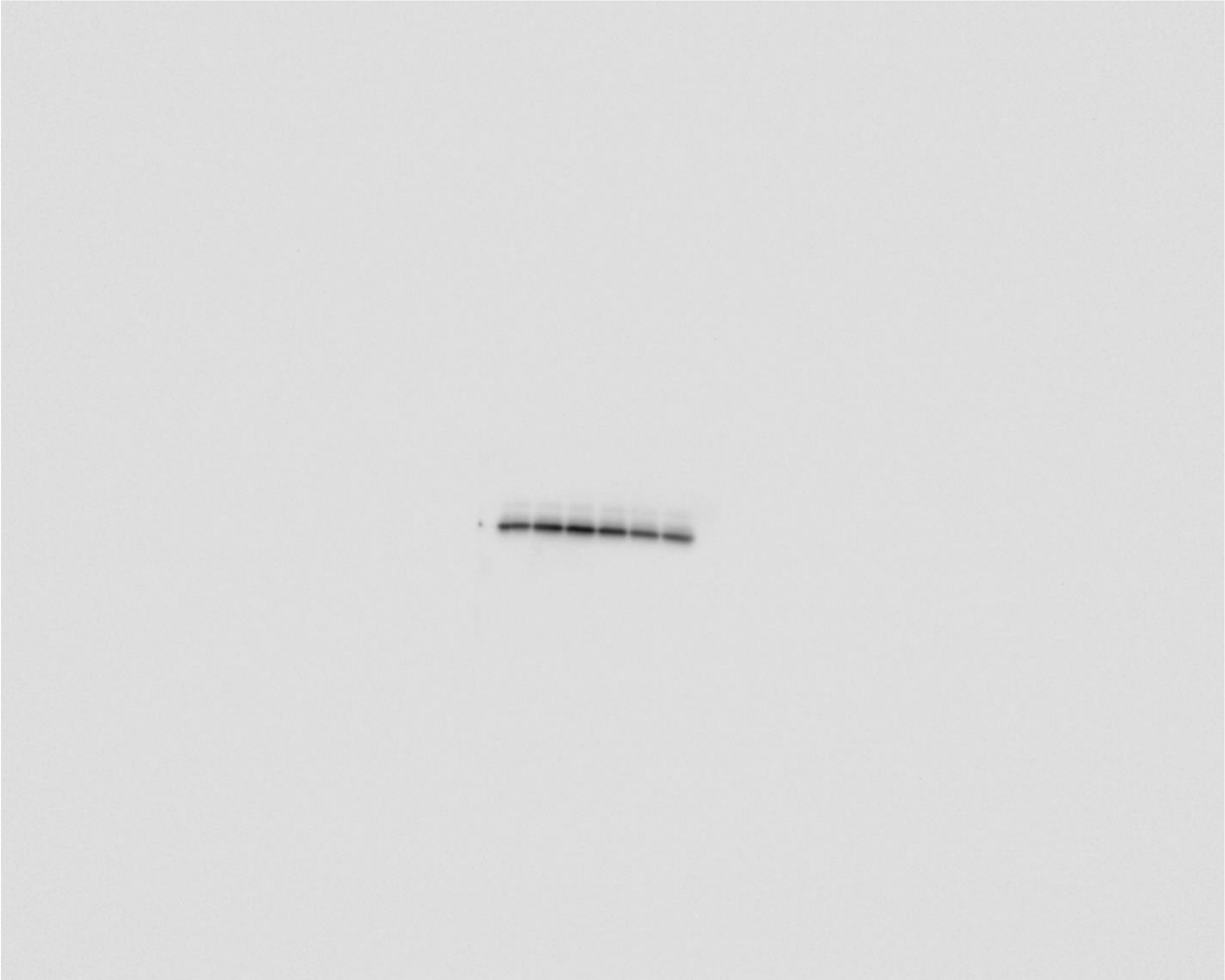

Figure 3E (HOS and OS921 cells):

tubulin:

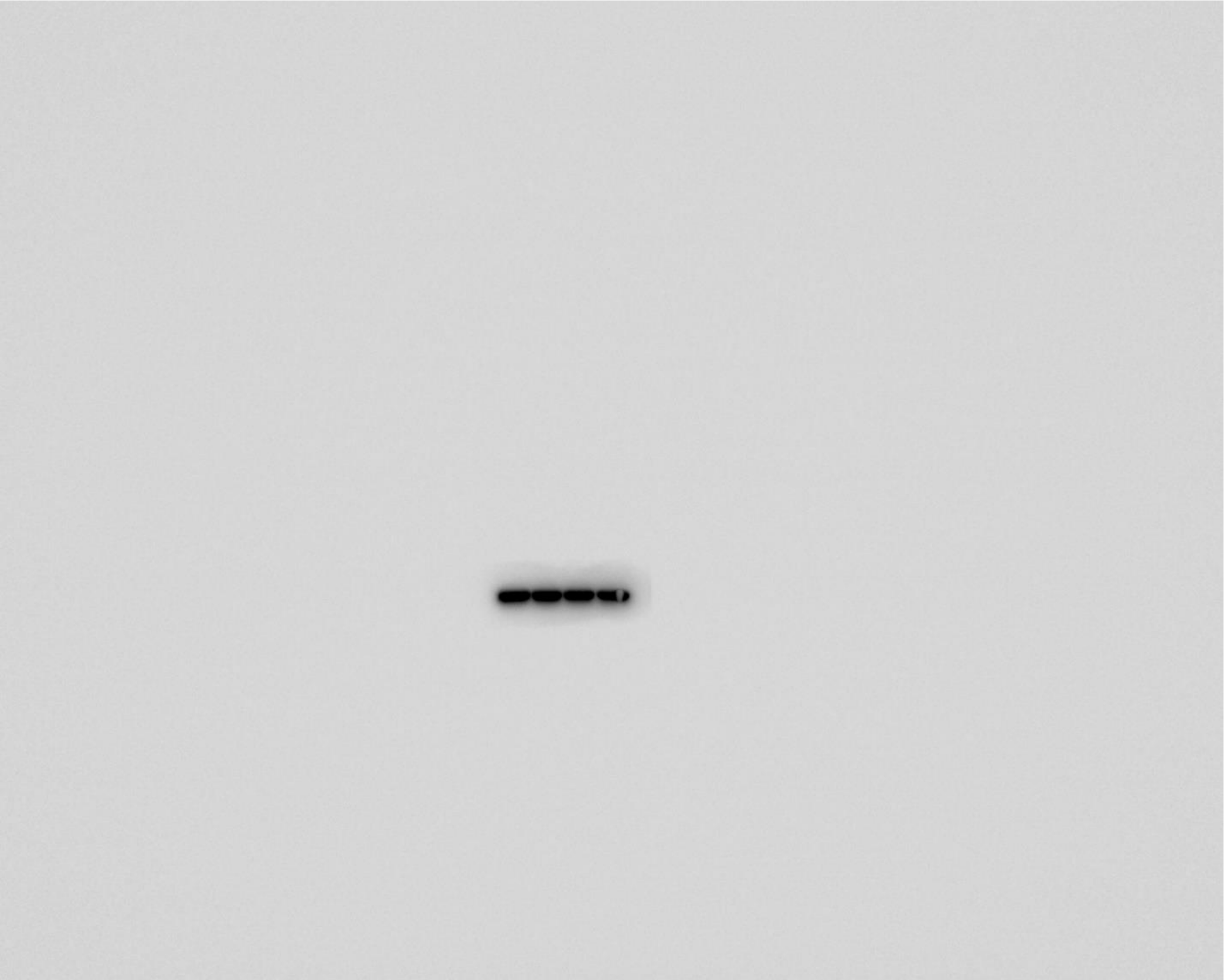

Figure 3E (HOS and OS921 cells):

Atg5:

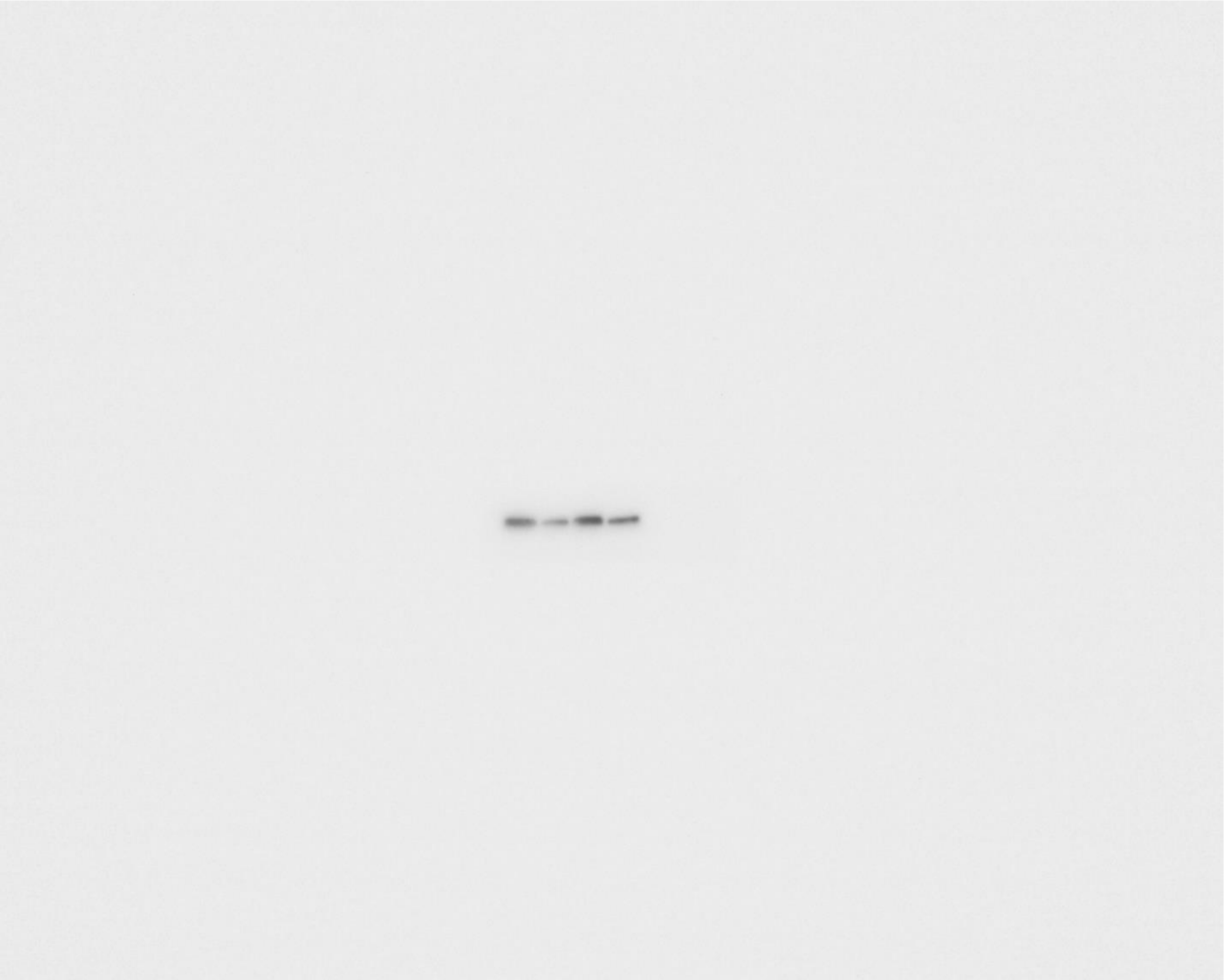

Figure 6H:

Atg5:

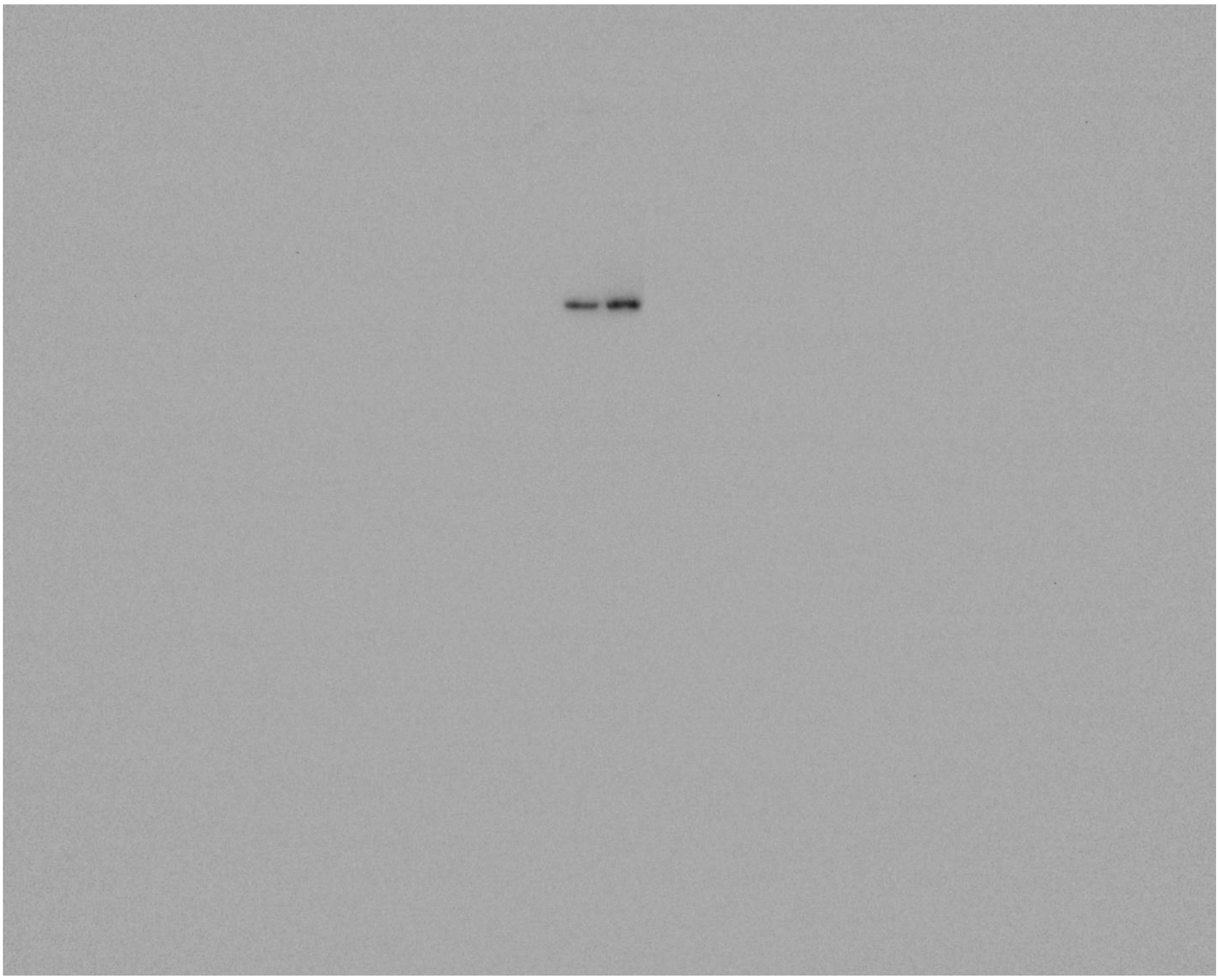

Figure 6H:

LC3 I/II:

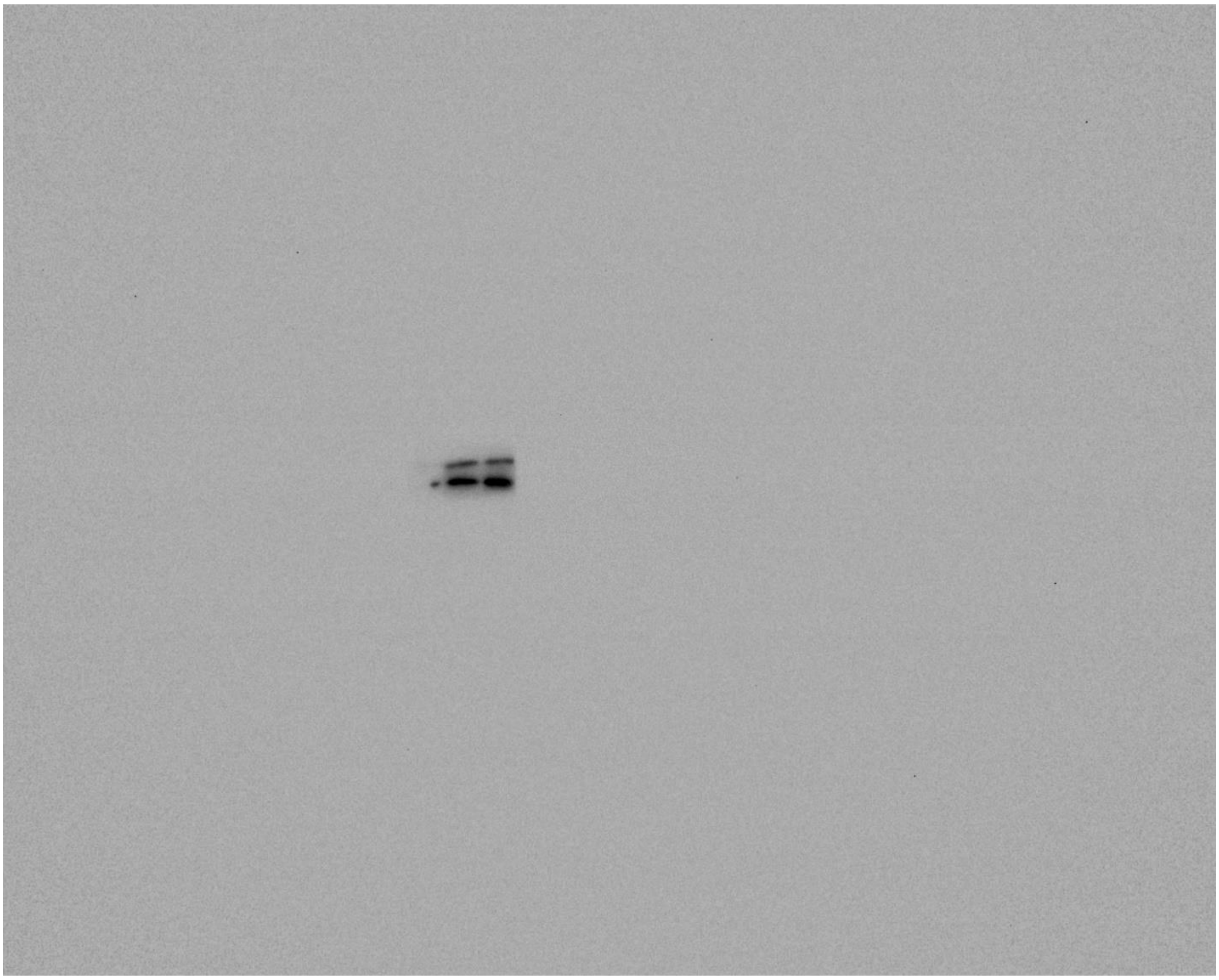

Figure 6H:

P62:

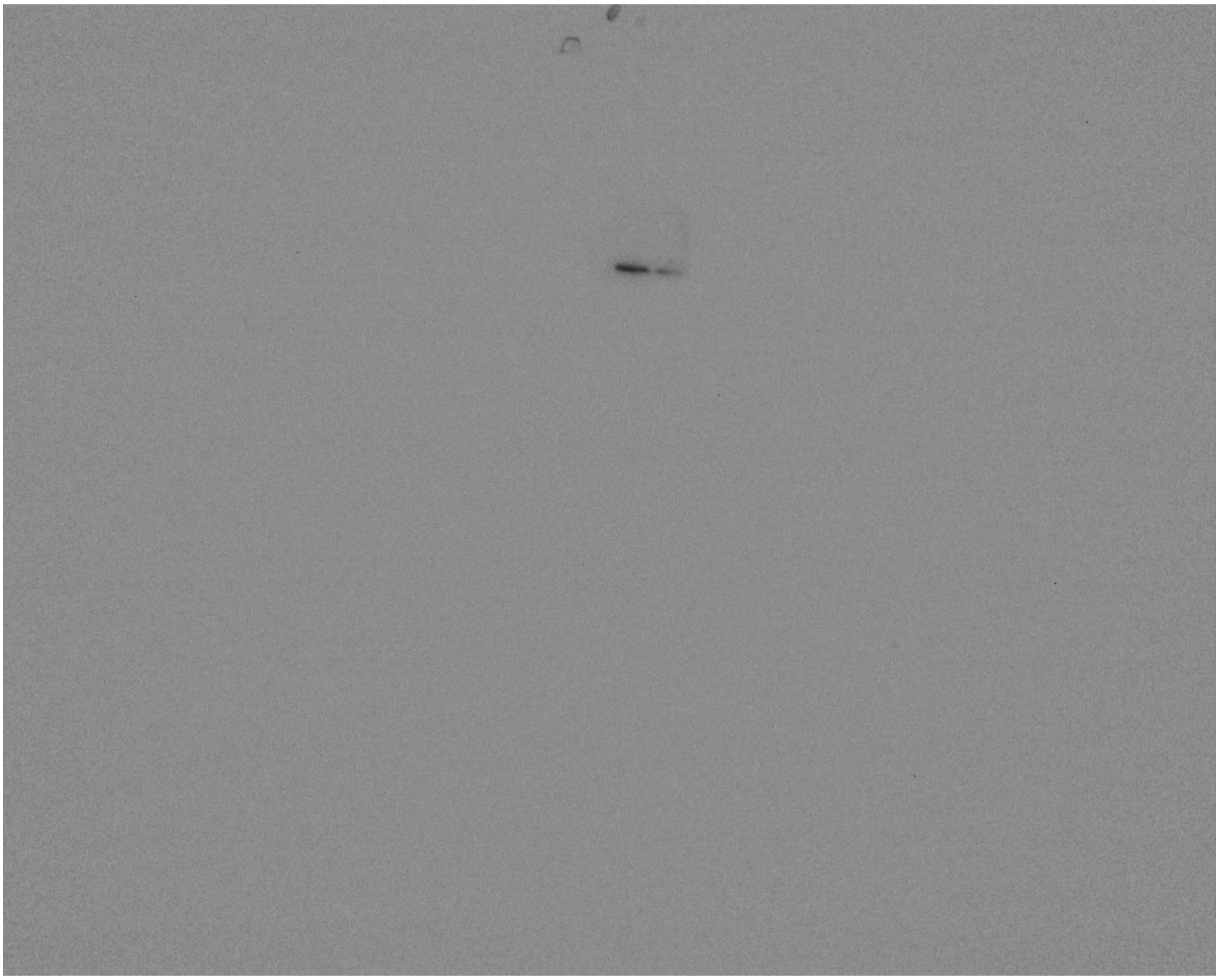

Figure 6H:

tubulin:

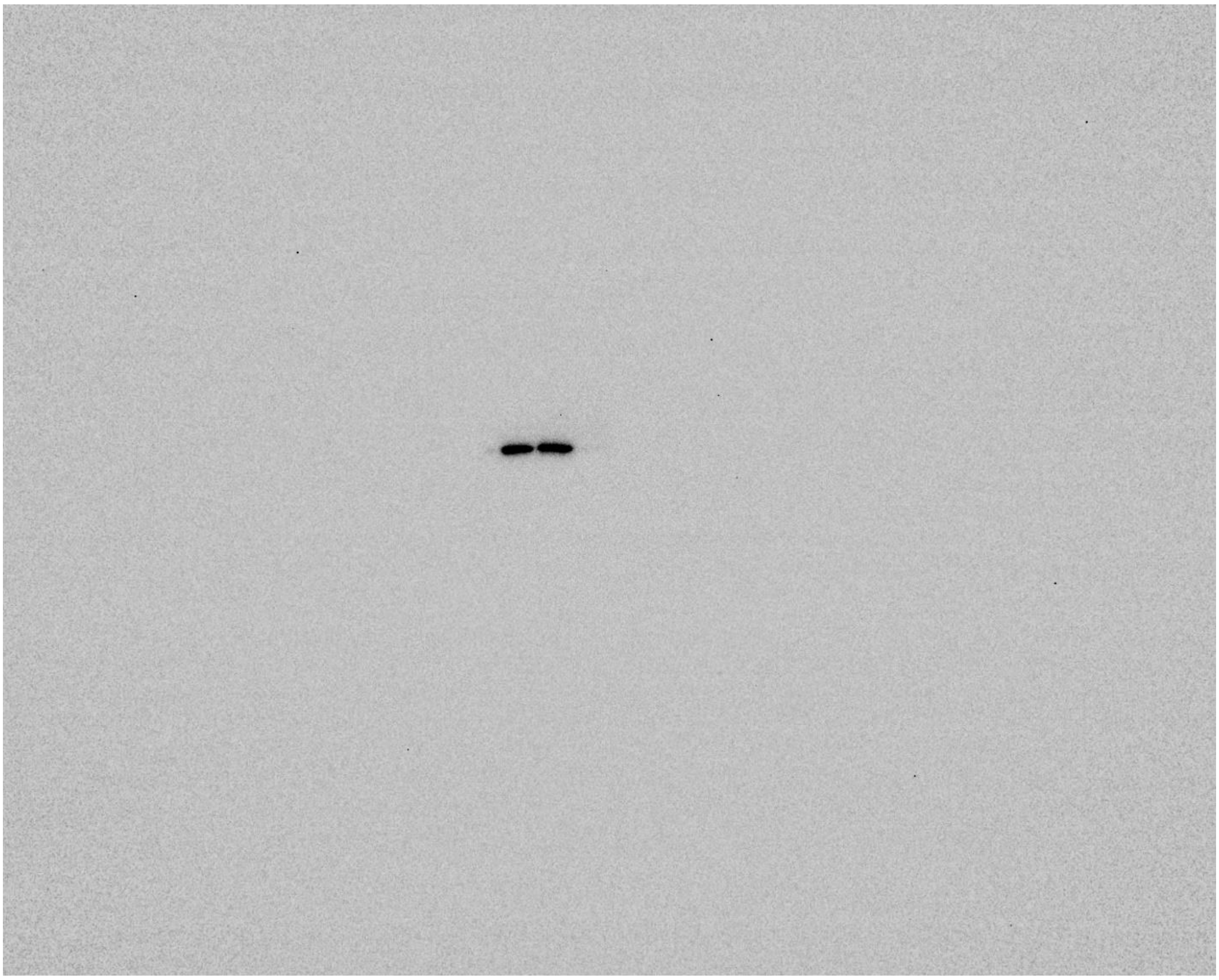

Supplement: Supplementary file 1 — Wet blots [file 41419_2025_8304_MOESM1_ESM.pdf]
