# Supplementary material for: Chloroquine Overcomes Chemotherapy Resistance and Suppresses Cancer Metastasis by Eradicating Dormant Cancer Cells
Source: Cell Death Dis. 2025 Dec 10;17(1):91. doi: 10.1038/s41419-025-08304-6 (PMC12830959; doi:10.1038/s41419-025-08304-6)
Supplement: Supplementary file 2 — Supplementary Material [file 41419_2025_8304_MOESM2_ESM.pdf]

Supplementary material to m/s “Chloroquine Overcomes Chemotherapy Resistance and Suppresses Cancer Metastasis by Eradicating Dormant Cancer Cells” by Mikeladze et al

Figure S1

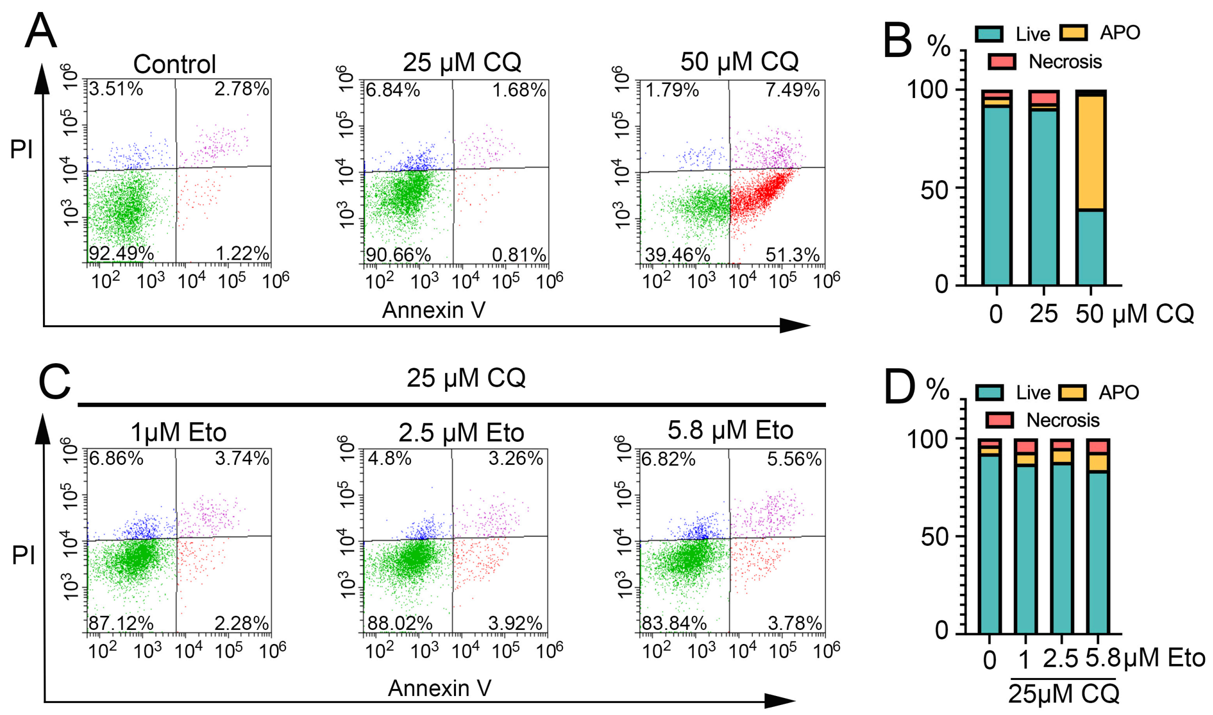

Figure S1. Chloroquine is well tolerated by normal fibroblasts (DF2) even when combined with etoposide

(A) Flow cytometry plots of A549 cells treated with 25 µM or 50 µM CQ. (B) Quantification of apoptotic cells from two independent experiments as in (A). (C) Flow cytometry plots of A549 cells treated with 25 µM CQ in combination with etoposide at the indicated concentrations. (D) Quantification of apoptotic cells from two independent experiments as in (C).

Figure S2

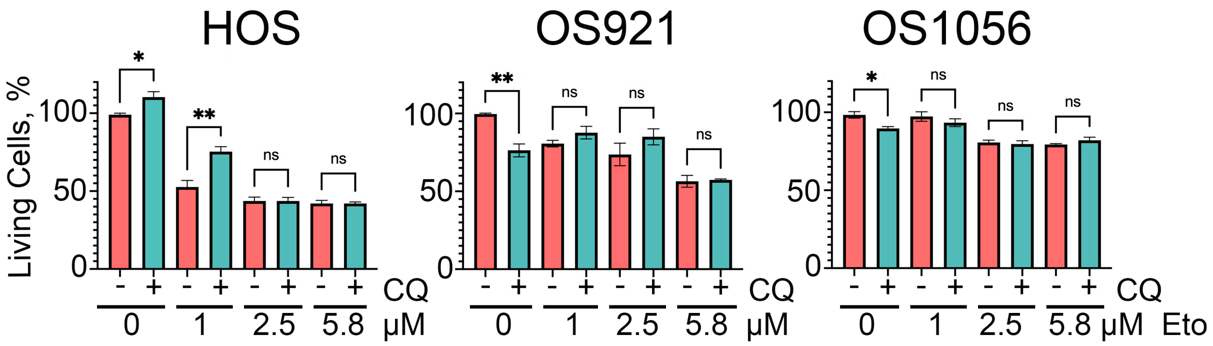

Figure S2. Simultaneous administration of etoposide and chloroquine does not enhance the cytotoxic effect of etoposide

HOS, OS921, and OS1056 cells were seeded in 96-well plates. The next day, following cell attachment, 25 µM CQ and etoposide at the indicated concentrations were added simultaneously. After 24 hours, cell viability was assessed using the MTT assay. *$p < 0.05$; **$p < 0.0001$.

Figure S3

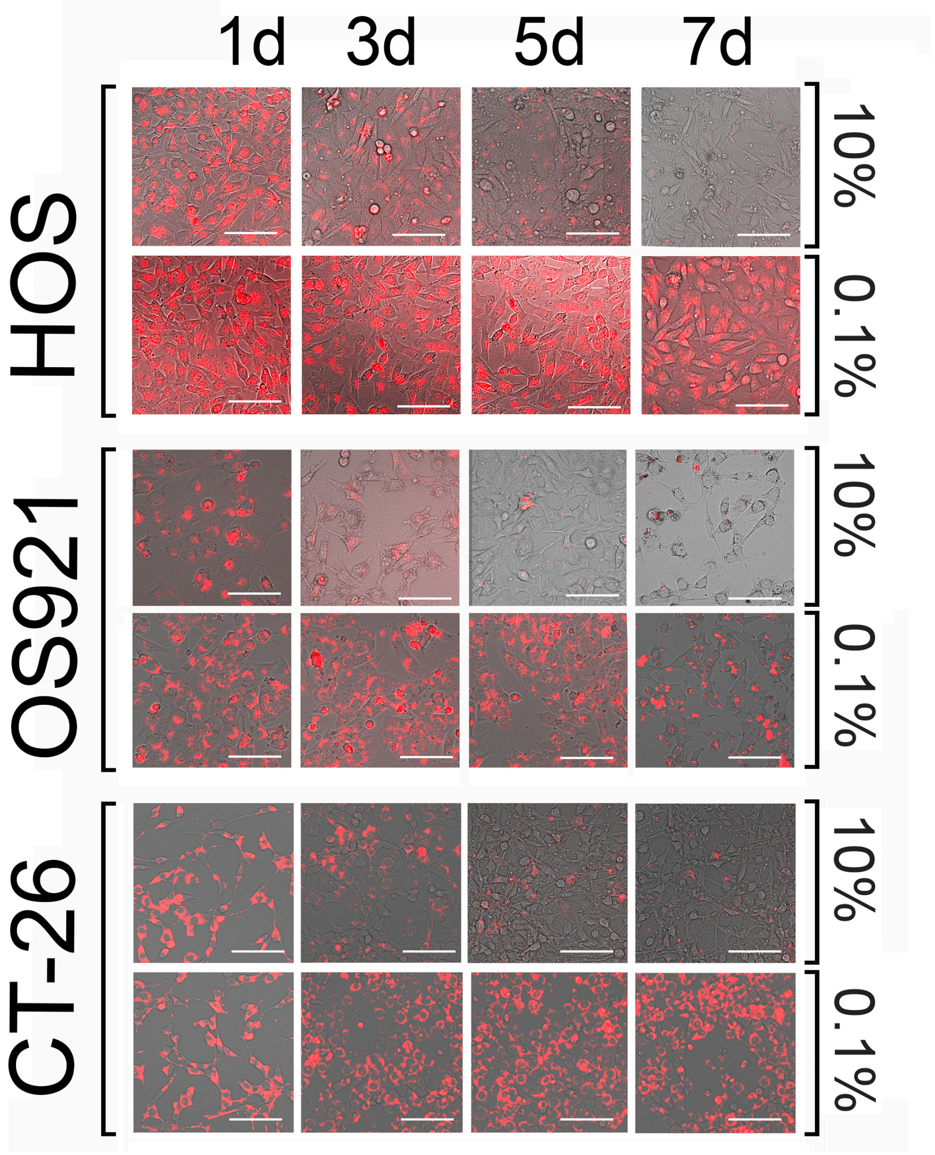

Figure S3. Dormancy model in osteosarcoma and CT-26 cells

HOS, OS921 and CT-26 cells were stained with PKH26 and seeded to wells of 12-well plates and the next day half of the wells were transfered to 0.1% FCS (day "0") and the wells were photographed. Photographing was repeated on days 3, 5 and 7. Cells that had stopped proliferating remained red, while proliferating cells lost their color with each division.
