# Supplementary figures and images for: Correction: Chloroquine overcomes chemotherapy resistance and suppresses cancer metastasis by eradicating dormant cancer cells
Source: Cell Death Dis. 2026 Apr 17;17(1):391. doi: 10.1038/s41419-026-08552-0 (PMC13090342; doi:10.1038/s41419-026-08552-0)

HOS

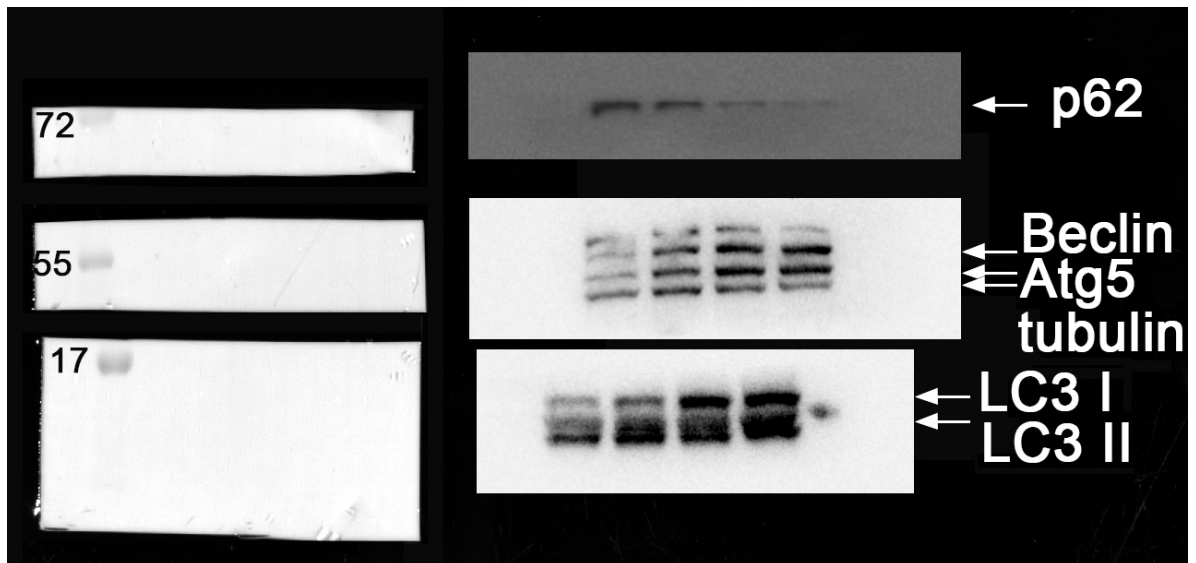

OS921

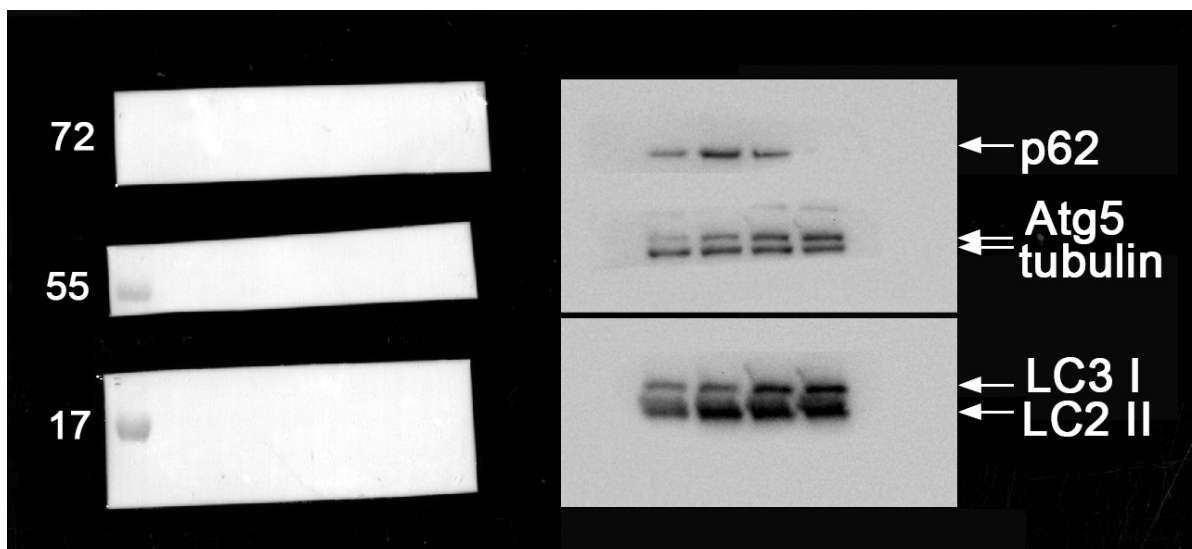

OS1056

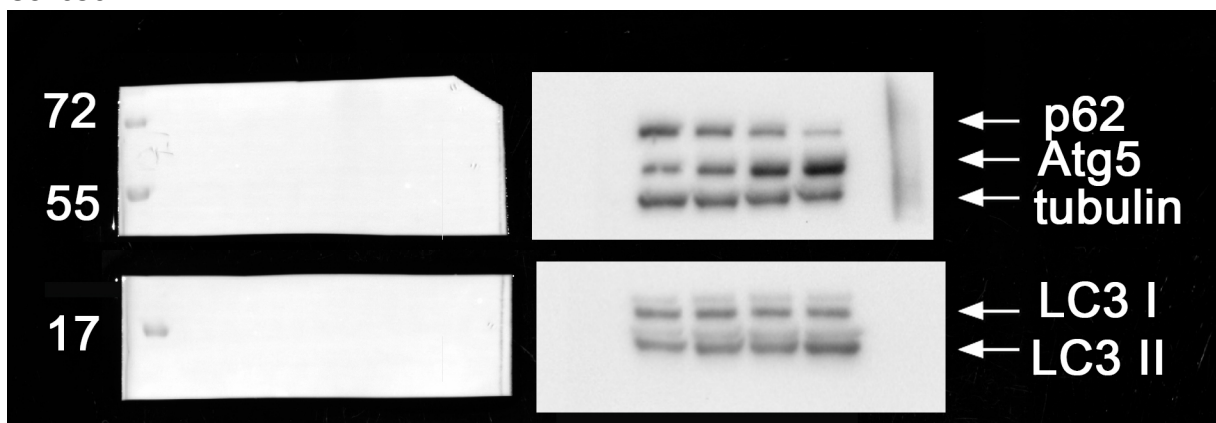

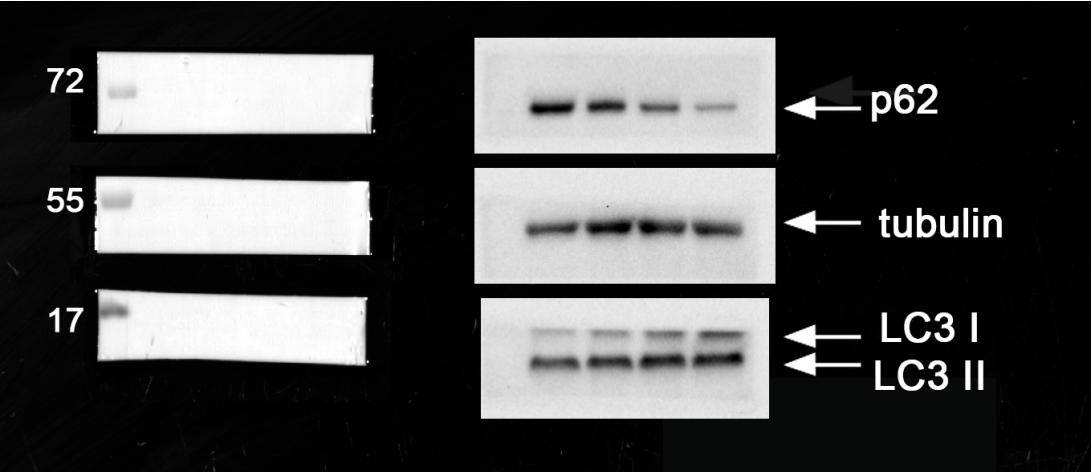

Supplement: Supplementary file 1 — Wet blots for erratum [file 41419_2026_8552_MOESM1_ESM.pdf]
